# A functional personalised oncology approach against metastatic colorectal cancer in matched patient derived organoids

**DOI:** 10.1038/s41698-024-00543-8

**Published:** 2024-02-27

**Authors:** Dexter Kai Hao Thng, Lissa Hooi, Bei En Siew, Kai-Yin Lee, Ian Jse-Wei Tan, Bettina Lieske, Norman Sihan Lin, Alfred Wei Chieh Kow, Shi Wang, Masturah Bte Mohd Abdul Rashid, Chermaine Ang, Jasmin Jia Min Koh, Tan Boon Toh, Ker-Kan Tan, Edward Kai-Hua Chow

**Affiliations:** 1 Cancer Science Institute of Singapore, National University of Singapore, Singapore, Singapore; 2 NUS Centre for Cancer Research (N2CR), Yong Loo Lin School of Medicine, National University of Singapore, Singapore, Singapore; 3 Department of Surgery, Yong Loo Lin School of Medicine, National University of Singapore, Singapore, Singapore; 4 Division of Colorectal Surgery, Department of Surgery, National University Hospital, National University Health System, Singapore, Singapore; 5 Division of Hepatobiliary & Pancreatic Surgery, Department of Surgery, National University Hospital, National University Health System, Singapore, Singapore; 6 Department of Pathology, National University Hospital, National University Health System, Singapore, Singapore; 7 Kyan Technologies, Singapore, Singapore; 8 The N.1 Institute for Health, National University of Singapore, Singapore, Singapore; 9 The Institute for Digital Medicine (WisDM), Yong Loo Lin School of Medicine, National University of Singapore, Singapore, Singapore; 10 Department of Pharmacology, Yong Loo Lin School of Medicine, National University of Singapore, Singapore, Singapore; 11 Department of Biomedical Engineering, College of Design and Engineering, National University of Singapore, Singapore, Singapore

**Keywords:** Colorectal cancer, Cancer, Translational research

## Abstract

Globally, colorectal cancer (CRC) is the third most frequently occurring cancer. Progression on to an advanced metastatic malignancy (metCRC) is often indicative of poor prognosis, as the 5-year survival rates of patients decline rapidly. Despite the availability of many systemic therapies for the management of metCRC, the long-term efficacies of these regimens are often hindered by the emergence of treatment resistance due to intratumoral and intertumoral heterogeneity. Furthermore, not all systemic therapies have associated biomarkers that can accurately predict patient responses. Hence, a functional personalised oncology (FPO) approach can enable the identification of patient-specific combinatorial vulnerabilities and synergistic combinations as effective treatment strategies. To this end, we established a panel of CRC patient-derived organoids (PDOs) as clinically relevant biological systems, of which three pairs of matched metCRC PDOs were derived from the primary sites (ptCRC) and metastatic lesions (mCRC). Histological and genomic characterisation of these PDOs demonstrated the preservation of histopathological and genetic features found in the parental tumours. Subsequent application of the phenotypic-analytical drug combination interrogation platform, Quadratic Phenotypic Optimisation Platform, in these pairs of PDOs identified patient-specific drug sensitivity profiles to epigenetic-based combination therapies. Most notably, matched PDOs from one patient exhibited differential sensitivity patterns to the rationally designed drug combinations despite being genetically similar. These findings collectively highlight the limitations of current genomic-driven precision medicine in guiding treatment strategies for metCRC patients. Instead, it suggests that epigenomic profiling and application of FPO could complement the identification of novel combinatorial vulnerabilities to target synchronous ptCRC and mCRC.

## Introduction

Globally, colorectal cancer (CRC) is the third most frequently occurring cancer, representing 10% of all newly diagnosed cancers in 2020^1^. At the point of diagnosis, 36% and 20% of all patients present regional spread and distant metastatic lesions (metastatic CRC [metCRC]) respectively^2^. Furthermore, 50% of patients with initially diagnosed localized disease will progress on to metCRC^3^. Progression of CRC from a localised lesion to an advanced metastatic malignancy is often indicative of poor prognosis in patients, as the 5-year survival rates declines rapidly from 90% to 10%^4^. In terms of the management of metCRC, the frontline treatment strategy for patients is systemic combination chemotherapy with either anti-EGFR targeted therapies or bevacizumab to mitigate the growth of primary and metastatic lesions concurrently^5^. However, metCRC patients often progress on mainstay chemotherapies with targeted biologics after a median duration of 12 to 18 months, with most developing resistance to FDA-approved systemic therapies within 30 months^6^. The poor prognosis for metCRC patients underscores the exploration of alternative treatment modalities for metCRC treatment, with a greater emphasis on combination therapies to circumvent the frequent emergence of tumour resistance. Furthermore, combination therapies enable the concurrent targeting of heterogeneous subclone populations with diverse vulnerabilities, improving the tumour coverage of systemic therapies^7^. This potentially increases the efficacy of treating the entire primary tumour mass together with synchronous metastases.

Intriguingly, there is a growing amount of evidence reflective of the interplay between genetic and epigenetic alterations in the progression of metCRC, suggesting that epigenomic changes can modulate treatment response^8^. Integrative genomic and epigenomic analyses have highlighted significant correlation between the DNA hypermethylation status of CRC patients and the mutational status of driver genes, *KRAS* and *BRAF*^9–12^. Notably, the genetic status of these genes are the main determinants of treatment strategies in metCRC patients, suggesting that the corresponding epigenomic profiles of patients may potentially modulate sensitivity to frontline targeted therapies. Correspondingly, clinical studies have delineated epigenetic signatures which could predict patient response to first-line bevacizumab and chemotherapy in a few cancer types, including breast and lung cancer^13–15^. Importantly, reversal of epigenetic reprogramming during disease progression with pharmacological inhibitors, such as histone deacetylase (HDAC) and DNA methyltransferase (DNMT) inhibitors, could overcome chemotherapy-resistance in tumour cells^16–19^. Noticeably, combining standard cancer therapies with epigenetic drugs have also been becoming increasingly popular, with greater success of such treatment modalities being observed preclinically and in the clinics^20^. Taken together, the existing literature highlights the emerging role of epigenetics in metCRC disease progression and treatment resistance, presenting a promising combination treatment modality for patients beyond the standards of care – possibly as subsequent lines of therapies in combination with chemotherapies.

Recent epigenomic profiling, however, revealed distinct methylation patterns associated with functional regulatory changes between metCRC primary tumour and metastases^21^. Coupled with the heterogeneous nature of tumour cell evolution during metastasis dissemination, we postulate that primary and metastatic lesions may respond differentially to the same epigenetic combination therapy^22,23^. There is thus a need to delineate the efficacy of epigenetic combination therapies across different lesion sites. Importantly, a greater appreciation for the inherently heterogeneous nature of the disease further necessitates the implementation of personalised medicine in tailoring the use of epigenetic combination therapy for the management of metCRC.

Many frequently used epigenetic therapies lack genomic or proteomic biomarkers to select patients who would potentially exhibit favourable treatment response. Hence, we propose a functional personalised oncology (FPO) approach in identifying tumour vulnerabilities by observing the phenotypic changes in tumour cells when subjected to genetic and pharmacological perturbations. Noticeably, with the protocol for the generation of patient-derived avatars of different cancers, including CRC, being established, there is a growing number of preclinical studies demonstrating the correlative concordance between ex vivo and clinical responses of tumours to the same therapies^24,25^. The preclinical evidence of the FPO approach has led to the conduct of several clinical trials worldwide, in which patient-derived avatars such as organoids and xenografts are used as functional drug screening systems to recommend the most appropriate treatment regimen for patients^26^. The FPO approach thus represents a tool with tremendous potential to change the current practice in which clinical decisions are made.

Here, we utilise the experimental-analytical hybrid drug combination interrogation platform, quadratic phenotypic optimization platform (QPOP), as a tool for FPO to identify the global optimal combination given a defined set of drugs, dosages and composition without any reference to the mechanisms of action of the drugs, or predetermined synergism^27^. Specifically, QPOP models the functional responses of biological systems to external perturbations using a second-order polynomial equation as prior studies had shown that other higher-order terms had negligible influence^28–30^. The utility of QPOP was shown in several studies, demonstrating its capabilities in providing clinical decision support for the management of haematological malignancies, as well as the identification of combinatorial vulnerabilities in solid tumours^27,31–33^. QPOP is therefore a potentially valuable FPO tool which could rapidly identify personalised combination therapies for the management of metCRC.

In elucidating the therapeutic potential of combinatorial systemic therapies, including epigenetic therapies, in metCRC, we thus leveraged on patient-derived organoids (PDOs) as clinically relevant models for FPO in this pilot study. To achieve this, a cohort of matched metCRC PDOs was established from the tumours of patients with synchronous metastases undergoing surgical resection. Subsequent functional drug screens allowed us to objectively discern the sensitivities of PDOs derived from primary tumours (ptCRC) and metastases (mCRC) to frontline and epigenetic systemic therapies alone and in combination, informing treatment strategies for metCRC patients. From this point, metCRC refers to the condition of the malignancy whilst mCRC refers specifically to only the metastatic lesions.

## Results

### Establishment of CRC organoids from primary tumours and metastases

In this study, clinically relevant models of CRC were generated from fresh patient tissues derived from primary tumour and metastatic sites of CRC. Twelve metCRC patients presenting synchronous primary tumours and metastases, and five non-metastatic CRC patients were enrolled in this study (Supplementary Table 1).

**Fig. 1:**
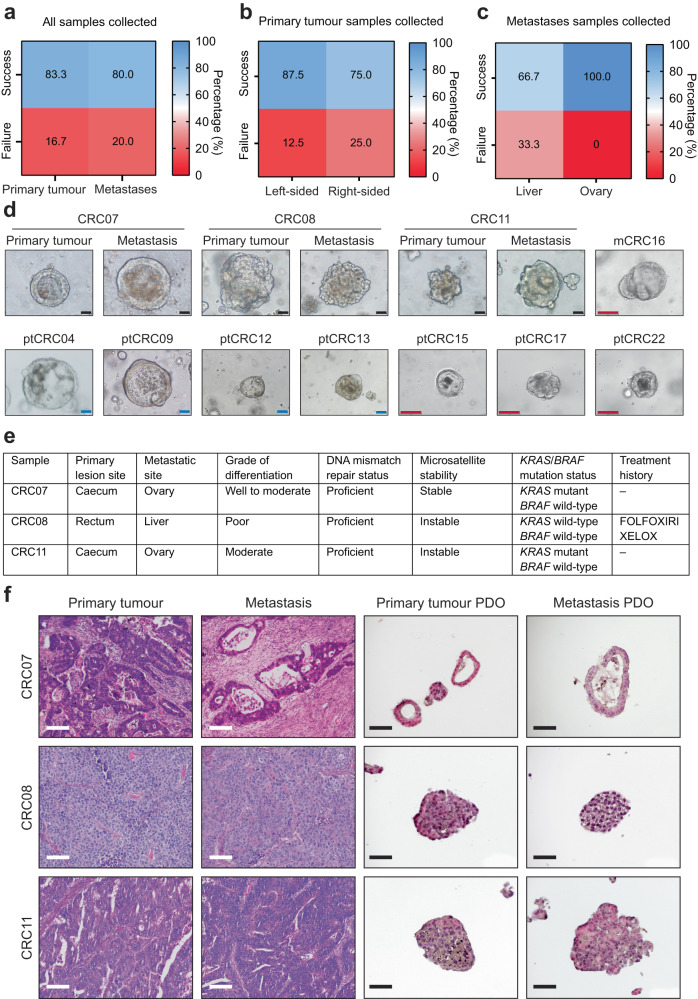
Establishment of metCRC patient-derived organoids recapitulates histological architecture of primary tissues. **a** Establishment rates of CRC PDOs from patient samples derived from primary and metastatic lesions. **b** Establishment rates of ptCRC PDOs based on tumour origin. **c** Establishment rates of mCRC PDOs stratified according to metastatic site. **d** Brightfield images of 14 generated CRC PDO lines, including three pairs of matched metCRC PDOs. Black scale bar = 50 µm; blue scale bar = 100 µm; red scale bar = 150 µm. **e** Summary of clinical data for metastatic colorectal cancer patients from which matched PDOs were established. (FOLFOXIRI, 5-fluorouracil + leucovorin + oxaliplatin + irinotecan; XELOX, capecitabine + oxaliplatin). **f** H&E staining of matched metCRC parental tumours and PDOs derived from the corresponding tissues. Black scale bar = 50 µm; white scale bar = 100 µm.

In establishing a living biobank of CRC PDOs using culture conditions described previously, we successfully cultured 10 primary tumour PDOs (83.3% overall success rate) and four CRC PDOs derived from metastases (80% overall success rate), which is in line with prior studies (Fig. 1a, Supplementary Table 1)^34,35^. While the origin of primary lesions did not affect the success rates of generating PDOs significantly, we observed greater success in PDO establishment from ovarian metastases compared to liver metastases, although it was a small patient cohort (Fig. 1b, c). In culture, the PDOs presented distinct morphological features, demonstrating the diverse nature of CRC tumours (Fig. 1d).

Of the 14 generated CRC PDOs, three pairs of matched metCRC PDOs (pt/mCRC07, pt/mCRC08 and pt/mCRC11) were established and used for subsequent downstream investigations and studies (Fig. 1e). Morphologically, the three pairs of matched organoids maintained the diversity in tumour architecture and histopathological features of the corresponding patient tissues (Fig. 1f). Luminal and cystic features were evident in CRC07 samples, while CRC08 and CRC11 samples were noticeably denser and had more compact structures. Furthermore, mCRC PDOs exhibited similar histopathological features to ptCRC PDOs, as in the patient tissues, suggesting that synchronous metastatic tumours retain the histopathological features of the primary tumours (Fig. 1f).

### metCRC organoids recapitulate tumour characteristics of parental tissues

**Fig. 2:**
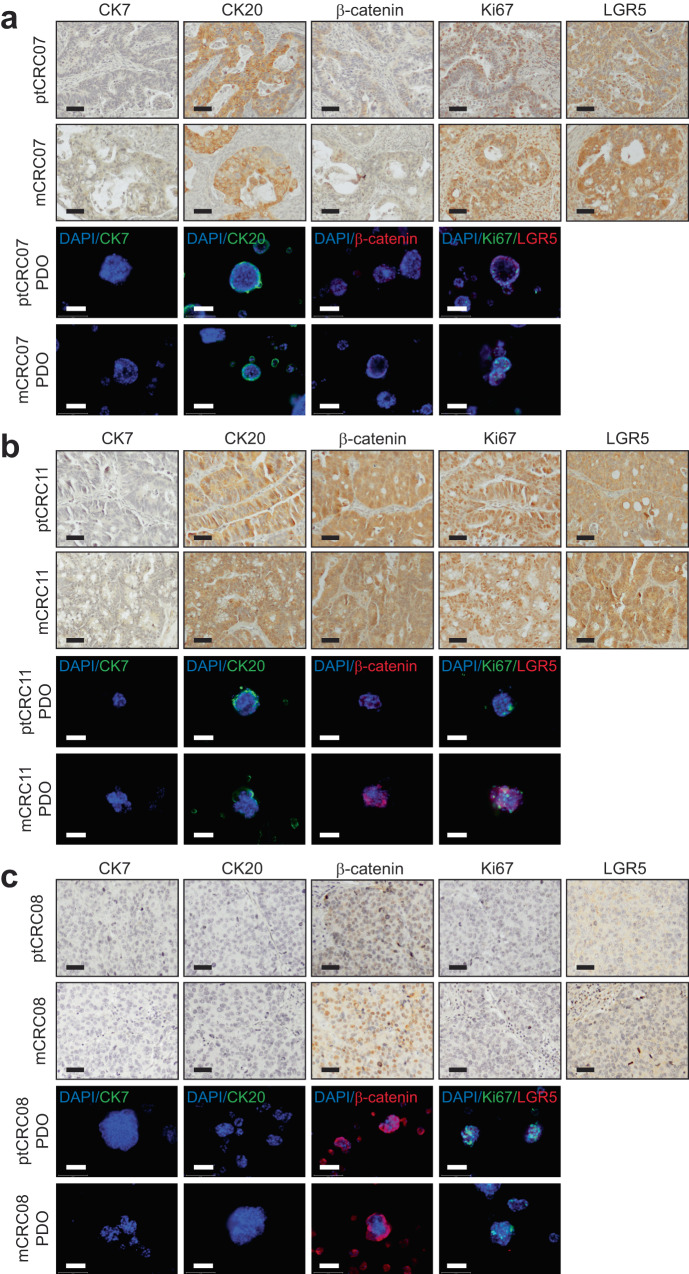
metCRC PDOs retain expression patterns of common CRC markers of parental tissues. **a** Immunohistochemistry staining of CK7, CK20, β-catenin, Ki67, and LGR5 in matched primary tumours and metastases, and immunofluorescent staining of the respective markers in the corresponding PDOs for three metCRC patients, CRC07, **b** CRC11 and **c** CRC08. Scale bars = 100 µm.

We proceeded to investigate the expression of important CRC molecular markers in the parental tumours and their respective PDOs for CRC07, CRC08 and CRC11. Importantly, the expression of CRC markers (CK7 and CK20), Ki67, and expression of CRC stem cell markers (β-catenin and LGR5) demonstrated strong concordance between the PDOs and tumour tissues (Fig. 2). CRC07 and CRC11 tumours and PDOs exhibited the CK7^–^/CK20^+^ phenotype, typical of colonic carcinoma (Fig. 2a, b)^36^. On the other hand, CRC08 tissues and PDOs demonstrated concurrent loss of both CK7 and CK20 (Fig. 2c). While the CK7^–^/CK20^–^ profile is less common in CRC tumours, prior evidence has demonstrated strong correlation between the loss of CK20 in poorly differentiated CRC tumours with microsatellite instability, consistent with the clinical characterisation of CRC08 tumours (Fig. 1e)^37,38^. Varied expression patterns of CRC markers β-catenin, LGR5 and Ki67 were observed across the patients, especially for β-catenin (Fig. 2). Interestingly, CRC08 tumours expressing the highest levels of nuclear β-catenin were derived from the hindgut, from which tumours conventionally present a higher incidence of *APC* mutations and hence enhanced nuclear β-catenin expression and localisation (Figs. 1e and 2c)^39^. Taken together, the strong concordance between the expression pattern of key CRC markers and clinical characteristics of the three metCRC patients support the robustness and reliability of the matched PDOs in recapitulating the tumour characteristics of the patients’ parental tumours.

To further characterise the expression of markers known to confer stem cell-like and self-renewal properties in organoids, the presence of cancer stem cell markers, Sox9, EpCAM and CD44, in the matched metCRC PDOs were assessed via immunofluorescence^40–42^. Expression of both Sox9 and EpCAM were retained across the matched PDOs from all three patients (Supplementary Fig. 1). However, CD44 was absent in CRC08 PDOs whilst present in CRC07 and CRC11 PDOs (Supplementary Fig. 1). The expression patterns of the various markers collectively demonstrated that midgut-derivative tumours, CRC07 and CRC11, generally have similar expression of CRC markers. On the contrary, expression of CRC markers were expectedly different in hindgut-derivative CRC08 tumours, which are characteristically different from midgut-derivative tumours (Fig. 1e)^39^. metCRC PDOs are therefore able to maintain the heterogeneous nature of CRC whilst preserving characteristics typical of the parental tissues.

### metCRC organoids preserve mutational spectrum of human colorectal cancer

**Fig. 3:**
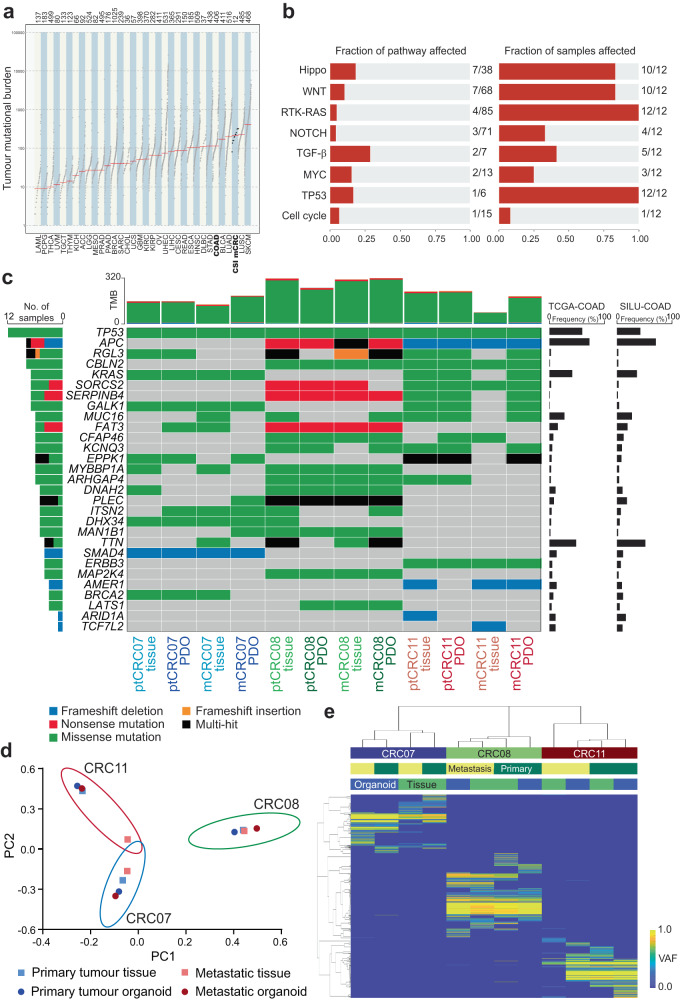
Mutational spectrum of CRC and parental tissues were represented in metCRC PDOs. **a** Tumour mutational burden of the matched metCRC tissues and PDOs used in this study (CSI-mCRC), and the GDC TCGA patient cohorts grouped by cancer type. CRC patient cohorts, GDC TCGA-COAD and CSI-mCRC, are bolded. **b** Frequency of genetic alterations found in common oncogenic pathways observed in our cohort of metCRC patients and corresponding PDOs. **c** Summary of most frequent mutations identified in pairs of metCRC parental tumours and PDOs. Representative genes known to be mutated in CRC are included. Mutation frequency of these genes in the GDC TCGA-COAD and Sidra–Leiden University Medical Center (SILU)-COAD cohorts are also presented for comparison. **d** Principal component analysis of patient tumours and PDOs according to the VAF of mutations present in the cohort. Closely related samples are grouped together. **e** Heatmap of patient tumour tissues and PDOs clustered by the VAF of 1199 unique single nucleotide polymorphisms and insertion/deletion mutations.

To explore the genetic representativeness of the three pairs of matched metCRC PDO models in our study, whole exome sequencing was performed in CRC07, CRC08 and CRC11 tumour tissues and PDOs. We found that the 12 metCRC samples in this study exhibited high tumour mutational burden (TMB), similar to the GDC TCGA-COAD patient cohort when compared across the spectrum of tumour types in the GDC TCGA dataset (Fig. 3a). Interrogations into components of 10 canonical oncogenic pathways as identified previously, revealed that genes related to signalling pathways frequently associated with CRC progression, including the Wnt, Hippo, Ras and p53 signalling pathways, were mutated in at least 10 of the 12 matched metCRC samples (83.3%) (Fig. 3b and Supplementary Table 2)^43^.

Mutational profiles of matched parental tumours and corresponding PDOs further recapitulated genetic alterations frequently observed in CRC, noticeably in key driver mutations of CRC, *TP53*, *APC* and *KRAS* (Fig. 3c)^44^. Genetic alterations were also observed in genes frequently mutated in CRC, including *TTN*, *SMAD4*, *ERBB3* and *MAP2K4* (Fig. 3c)^34,45–47^. Interestingly, several genetic mutations which were not common in prior CRC patient cohorts were found in our cohort, such as *RGL3* and *SERPINB4*, although this was attributed to the small sample size of three patients (Fig. 3c)^45,48^. Importantly, mutational profiles and TMB of PDOs were highly similar to their respective parental tumours, highlighting the fidelity of the established PDOs in retaining the genetic variants in the tumour tissues, with the exception of mCRC11 primary tissue (Fig. 3c). However, the mCRC11 tumour still harboured early-acquired CRC driver mutations in *TP53* and *APC*, suggesting that the tissue section sequenced likely contained a mixture of non-malignant tissue surrounding the tumour amidst the tumour section (Fig. 3c)^44^.

Principal component and cluster analysis were additionally performed based on the variant allele frequencies (VAF) of 1199 unique single nucleotide variants and mutations detected in the cohort to determine how closely the 12 samples relate to each other (Fig. 3d, e). Both analyses demonstrated that the parental tumours and PDOs originating from the same patient were grouped more closely together, suggesting that the CRC metastases in our cohort are genetically similar to the primary tumour for the most part (Fig. 3d, e). Collectively, our sequencing analyses demonstrated the ability of metCRC PDOs in recapitulating the mutational spectrum of the disease and preserving the genetic characteristics of parental tumours.

Phylogenetic analysis of the exome sequencing data was subsequently performed to elucidate the genetic stability of metCRC PDOs from the patient tumours. Mutations in early drivers of CRC, *TP53*, *APC* and *KRAS*, as well as in genes frequently altered in CRC, *FAT3*, *SMAD4*, *MAP2K4* and *ERBB3*, were found to be shared between parental tumours and PDOs (Supplementary Fig. 2a)^34,45–47^. However, PDOs acquired and lost unique mutation patterns such as in *BRCA2* (CRC07) and *AMER1* (CRC11), possibly due to clonal evolution or selection of the PDOs in culture (Supplementary Fig. 2a). As the mutational profiles differed between the three metCRC patients, the overlap in unique shared and non-shared mutations was determined and used to plot the phylogenetic pattern for each respective patient (Supplementary Fig. 2b). Noticeably, the patterns of linear and branched evolution elucidated were distinct between patients, with PDOs branching off from parental tumours in CRC07 samples while metastatic tumours branched from primary tumours in CRC08 (Supplementary Fig. 2ci, cii). The varied pattern of tumour evolution for CRC11 was also likely attributable to the nature of the mCRC11 tissue (Supplementary Fig. 2ciii). The results therefore reflect the heterogeneous nature of the disease, while indicating that metCRC PDOs can readily change in culture through the acquisition and loss of genetic alterations. Downstream functional studies were thus performed in early passages (<15) of metCRC PDOs to ensure that genomic features of metCRC PDOs closely resembled the parental tumours.

### CRC organoids exhibit similar sensitivities to anti-cancer agents

To evaluate the utility of CRC PDOs as in vitro drug screening systems, dose-response assays of 10 cancer therapeutics were performed in the first 10 PDO lines established, including the three pairs of matched metCRC PDOs. These included five standard of care therapies, 5-fluorouracil, oxaliplatin, leucovorin, SN-38 (the active metabolite of irinotecan), and regorafenib, two pharmacological inhibitors of arginine methyltransferases 4 (PRMT4) and 5 (PRMT5), TP-064 and pemrametostat respectively, and three epigenetic therapies currently employed in clinical trials against CRC, decitabine (NCT01193517), entinostat (NCT01105377) and vorinostat (NCT00942266). Notably, the panel of CRC PDOs exhibited varied responses to the same pharmacological agents (Table 1 and Supplementary Figure 3). The sensitivities of the PDOs generally fell within a range similar to the maximum serum concentration clinically attainable (*C*_max_), except for epigenetic drugs, pemrametostat, TP-064 and decitabine (Table 1).

**Table 1:** Summary of IC_50_ values for 10 drugs derived from single-drug dose-response assays in 10 CRC PDO lines

Drug	Average IC_50_ (µM)										CRC PDO Average IC_50_ (µM)	C_max_ (µM)
	ptCRC04	ptCRC07	mCRC07	ptCRC08	mCRC08	ptCRC09	ptCRC11	mCRC11	ptCRC13	mCRC16		
5-fluorouracil	8.66 ± 8.32	36.72 ± 15.1	18.54 ± 13.18	11.62 ± 4.05	69.63 ± 47.43	1.09 ± 0.23	194 ± 32.13	17.94 ± 9.58	46.51 ± 25.44	127.57 ± 24.1	53.23	426
Oxaliplatin	8.85 ± 5.9	24.55 ± 13.58	10.97 ± 1.36	2.62 ± 1.67	22.11 ± 9.78	1.04 ± 1.18	120.97 ± 32.18	10.28 ± 1.79	37.15 ± 22.62	14.26 ± 9.33	25.28	4.96
Leucovorin	N.D.	N.D.	N.D.	N.D.	N.D.	N.D.	N.D.	N.D.	N.D.	N.D.	N.D.	2.86
SN-38	0.00528 ± 0.00071	0.01656 ± 0.01551	0.00514 ± 0.0026	0.008 ± 0.002	0.2806 ± 0.19554	0.0059 ± 0.0003	0.37023 ± 0.03774	0.13 ± 0.18	0.16982 ± 0.16458	0.4289 ± 0.29754	0.14204	0.143
Regorafenib	8.66 ± 4.34	7.23 ± 4.83	2.64 ± 0.28	2.24 ± 0.98	2.24 ± 0.28	5.4 ± 3.25	9.34 ± 2.89	3.78 ± 1.05	7.85 ± 3.67	6.42 ± 4.47	5.58	8.08
Pemrametostat	285.2 ± 156	214.87 ± 169.81	5.61 ± 3.22	67.99 ± 46.55	89.82 ± 53.4	42.75 ± 35.17	N.D.	N.D.	160.15 ± 98.28	N.D.	123.77	3.54
TP-064	18.06 ± 9.02	8.38 ± 3.34	3.05 ± 1.05	15.83 ± 4.13	24.02 ± 5.95	10.72 ± 0.57	22.6 ± 13.02	6.92 ± 3.18	47.15 ± 11.18	38.14 ± 5.38	19.49	–
Decitabine	2.26 ± 2.43	48.17 ± 38.73	25.98 ± 16.58	133 ± 110.65	207.93 ± 16.46	18.53 ± 25.67	149.92 ± 122.97	251.88 ± 170.47	395.33 ± 252.84	N.D.	113.62	0.323
Entinostat	2.29 ± 1.79	3.17 ± 1.43	28.7 ± 1.41	1.11 ± 0.06	1.26 ± 0.3	0.73 ± 0.31	0.73 ± 0.12	5.26 ± 2.97	3.19 ± 1.09	1.51 ± 0.24	4.80	0.19
Vorinostat	0.67 ± 0.32	1.85 ± 0.7	1.27 ± 0.23	0.998 ± 0.179	0.63 ± 0.28	1.01 ± 1.46	1.07 ± 0.35	2.75 ± 1.19	1.43 ± 0.42	0.24 ± 0.08	1.19	1.2

IC_50_ values are represented as means ± SD (*n* = 3). *C*_max_ concentrations were determined from prior pharmacokinetic studies. *C*_max_ for TP-064 has not yet been established.

*N.D.* not determined.

**Fig. 4:**
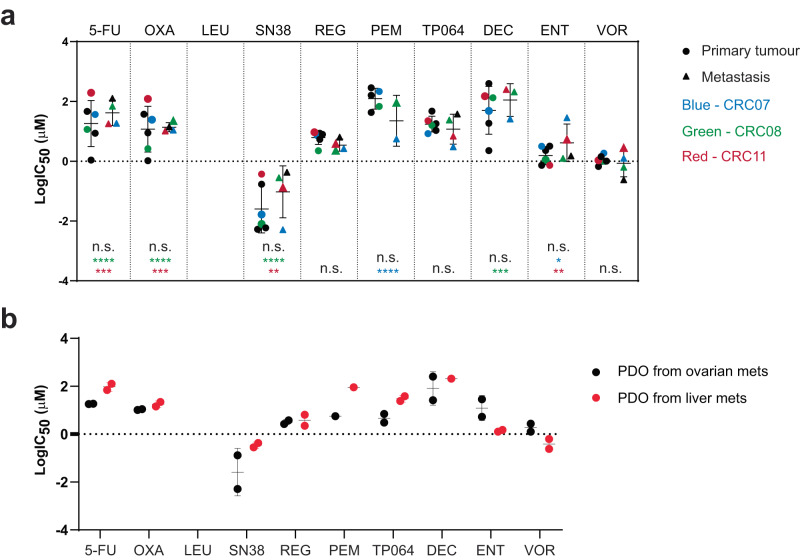
metCRC PDOs demonstrate similar drug responses. **a** Summary of IC_50_ values when PDOs were grouped according to tumour type. Matched PDOs were highlighted in blue (CRC07), green (CRC08) and red (CRC11). Two-way ANOVA and Šidák’s pairwise comparisons were performed as recommended (n.s. not significant; *p < 0.05; **p < 0.01; ***p < 0.001; ****p < 0.0001). Data represented as means ± SD (*n* = 6 and 4 for primary tumour and metastasis PDOs respectively). **b** Overview of IC_50_ when metastases PDOs were stratified based on metastatic site. Two-way ANOVA and Šidák’s pairwise comparisons were performed as recommended, but no statistical difference was identified between liver and ovarian metastases-derived PDOs for all 10 drugs. Data represented as means ± SD (*n* = 2). (5-FU 5-fluorouracil, OXA oxaliplatin, LEU leucovorin, SN38 SN-38, REG regorafenib, PEM pemrametostat, TP064 TP-064, DEC decitabine, ENT entinostat, VOR vorinostat).

Comparisons between PDOs derived from the primary tumours and metastases, also revealed no significant differences in their response to the panel of drugs (Fig. 4a and Supplementary Fig. 4a). Notably, patient CRC08 had received and progressed on several lines of chemotherapy regimens prior to the concurrent resection of synchronous primary tumour and liver metastases, including FOLFOXIRI (5-fluorouracil + leucovorin + oxaliplatin + irinotecan) and XELOX (capecitabine + oxaliplatin) (Fig. 1e). Correspondingly, mCRC08 exhibited greater resistance than ptCRC08 to these therapeutics, especially in SN-38, demonstrating the progression of metastases on systemic chemotherapies and the concordance between the drug sensitivities of the patient and PDOs.

As most drugs are frequently metabolised in the liver, we additionally compared the sensitivities of liver and ovarian mCRC PDOs to assess if drug metabolism would influence the sensitivity of liver metastases to the same drugs. Furthermore, a clinical trial in metCRC patients previously reported a greater resistance of liver metastases to chemotherapies when compared to lung metastases^49^. However, PDOs from liver metastases in our cohort (mCRC08 and mCRC16) exhibited minimal resistance to eight of the drugs when compared to PDOs from the ovarian metastases (mCRC07 and mCRC11), with only SN-38 and TP-064 presenting a statistically significant higher AUC values (Fig. 4b and Supplementary Fig. 4b). The data therefore suggest that while the mCRC PDOs in our study can recapitulate the genetic diversity of the disease, they respond similarly to anti-cancer agents for the large part when used singly, albeit being a small patient cohort with different treatment history.

### QPOP identified organoid-specific combinatorial vulnerabilities in matched metCRC organoids

As we have demonstrated that CRC PDOs were amenable to drug screens and given the high incidence of tumour resistance to monotherapies, we then sought to evaluate if concurrent delivery of the drugs in combination would enhance their therapeutic value and increase its coverage in matched metCRC PDOs. To rationally design optimal drug combinations effective against matched metCRC PDOs, we leveraged on the phenotypic-driven platform, QPOP. Phenotypic response of the PDOs to the 91 combinations derived from the resolution IV OACD were used as the input variables for QPOP analyses and the generation of a second-order linear regression model to determine the projected viabilities of PDOs to all drug combinations within the search space for the panel of drugs (Supplementary Tables 3–5)^50^. QPOP analyses identified tumour-specific combinatorial vulnerabilities, as the top-ranked two-drug combinations differed across all six metCRC PDO lines (Supplementary Fig. 5). However, concurrent delivery of four systemic therapies to target both the primary tumour and metastases is uncommon due to the likelihood of drug-induced toxicities. Hence, the expected viabilities of the PDOs to all two-drug combinations were analysed collectively to determine whether QPOP could rationally design two-drug combinations to target both ptCRC and mCRC PDOs concurrently.

**Fig. 5:**
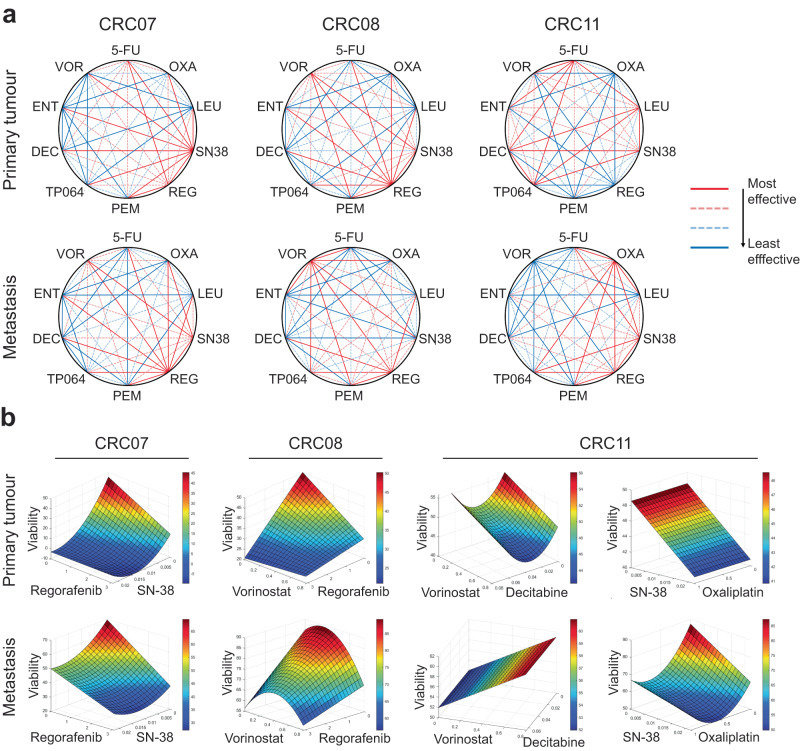
Matched metCRC PDOs exhibit differential sensitivity patterns to QPOP-optimised drug combinations. **a** Representative polygonograms depicting the effects of all two-drug combinations between 5-fluorouracil (5-FU), oxaliplatin (OXA), leucovorin (LEU), SN-38 (SN38), regorafenib (REG), pemrametostat (PEM), TP-064 (TP064), decitabine (DEC), entinostat (ENT) and vorinostat (VOR) in matched metCRC PDOs (*n* = 2; Supplementary Fig. 6). The coloured lines represent the ranked percentile of the geometric means for the viabilities of the PDOs in response to each combination. (Red, most effective, 75–100th percentile; pink and dotted, second most effective, 50–75th percentile; light blue and dotted, second least effective, 25–50th percentile; dark blue, least effective, 0–25th percentile). **b** Representative parabolic response surface maps illustrating the projected efficacy of the QPOP-optimised drug pairs on organoid viability for each respective pair of matched metCRC PDOs (*n* = 2; Supplementary Fig. 6).

Comparative analyses of all two-drug combinations based on the QPOP-derived viabilities in matched metCRC PDOs revealed distinct sensitivity patterns between patients and differential responses to the panel of drugs in combination. CRC07 PDOs were collectively sensitive to the concurrent inhibition of regorafenib and SN-38, while CRC08 PDOs were more responsive to regorafenib in combination with vorinostat (Fig. 5a). Noticeably, metastases-derived PDOs exhibited similar drug combination vulnerabilities as the primary tumour-derived PDOs in both patients, suggesting that a single drug combination was sufficient in targeting both the primary and metastatic tumour for both patients (Fig. 5a). Conceivably, similar drug-drug interactions were observed in response surface maps for the corresponding drug combinations in the matched ptCRC and mCRC PDO of both patients, with a decrease in organoid viability with increasing concentrations of both drugs (Fig. 5b).

Importantly, ptCRC11 and mCRC11 PDOs presented differential sensitivity patterns to the two-drug combinations despite sharing significant genetic similarities. ptCRC11 was more responsive to epigenetic-based drug combinations but exhibited greater resistance to combinations comprising of frontline therapies such as oxaliplatin, SN-38 and regorafenib (Fig. 5a). Conversely, the opposite was observed in mCRC11 (Fig. 5a). Specifically, decitabine in combination with vorinostat was shown to be effective in only ptCRC11, whilst exhibiting antagonistic interactions in mCRC11 (Fig. 5b). On the other hand, ptCRC11 was expected to be unresponsive to oxaliplatin despite demonstrating strong efficacy in mCRC11 (Fig. 5b). Taken together, the QPOP analyses exemplified the diverse sensitivity patterns of metCRC primary tumours and metastases in response to combination therapies which may not be apparent in single-drug sensitivity assays and genetic profiling.

### Matched organoids as tools for FPO in metCRC

**Fig. 6:**
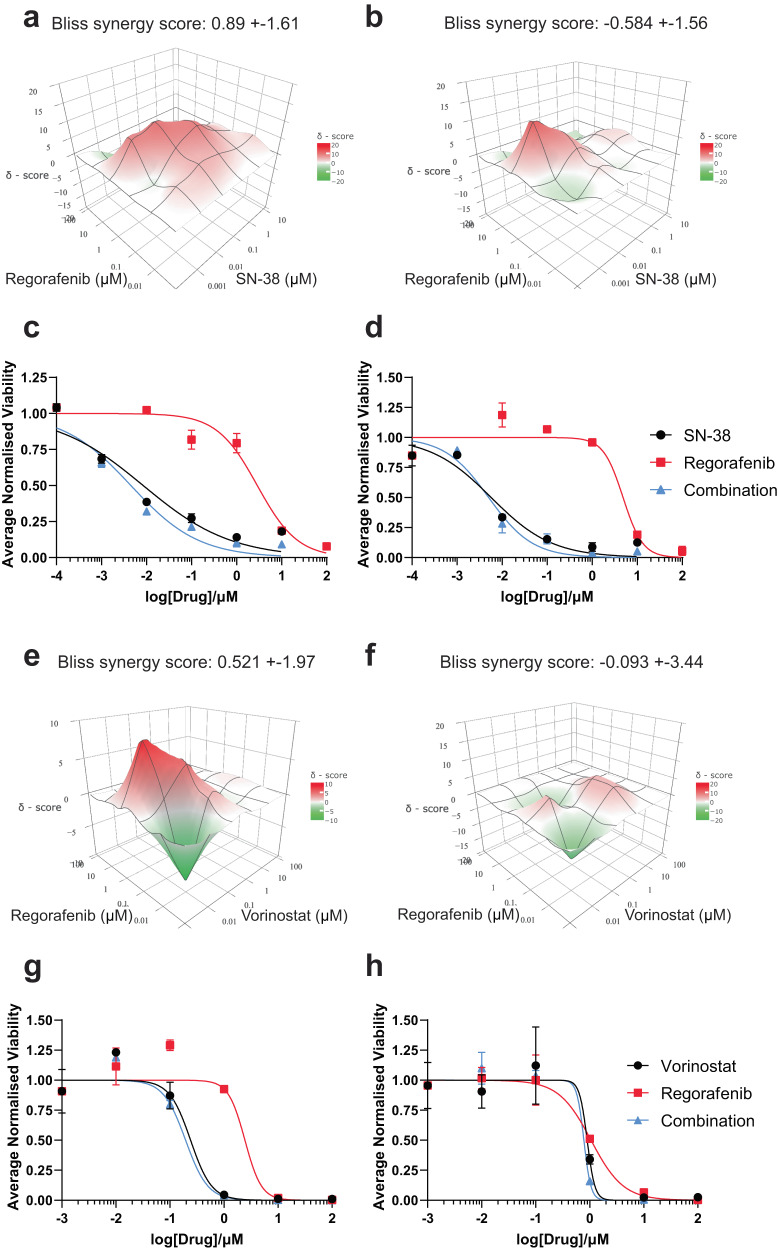
CRC07 and CRC08 PDOs were sensitive to regorafenib in combination with SN-38 and vorinostat combination therapy respectively. **a** Bliss synergy maps representing the synergy landscape for regorafenib-SN-38 in ptCRC07 and **b** mCRC07 over the entire dose matrix. Bliss synergy scores are represented as means of the entire search space ± 95% confidence interval. **c** Dose-response curves of regorafenib and SN-38 for ptCRC07 and **d** mCRC07 PDOs when treated as monotherapies and combination therapy. **e** Bliss synergy maps for regorafenib in combination with vorinostat in ptCRC08 and **f** mCRC08 over the entire dose matrix. Bliss synergy scores are represented as means of the entire search space ± 95% confidence interval. **g** Dose-response curves of ptCRC08 and **h** mCRC08 organoids when treated with regorafenib and vorinostat singly and in combination.

The matched metCRC PDOs were treated with their respective QPOP-optimised drug combinations at varying concentrations to validate their efficacy as personalised combination therapies. As predicted by the QPOP analysis, CRC07 PDOs responded similarly to the regorafenib-SN-38 dose matrix, as increasing concentrations of both drugs reduced the viabilities of the PDOs considerably (Supplementary Fig. 7). The bliss synergy score of the PDO responses to the drug pair was indicative of an additive interaction (−10 < *δ* < 10), with the combination exerting similar effects in both PDO lines (Fig. 6a, b). Individual dose-response curves for regorafenib and SN-38 as monotherapies and in combination additionally demonstrated a slight leftwards shift in the curve for the drug combination, supporting the additive nature of the drug pair (Fig. 6c, d). Morphological changes in PDOs were monitored to trace the organoids’ phenotypic responses. CRC07 PDOs gradually decreased in size with greater disruptions in structure as drug dosages progressively increased, especially for the drug combination (Supplementary Fig. 8a).

Similar trends were observed in CRC08 PDOs treated with regorafenib in combination with vorinostat. Treatment of CRC08 PDOs with the regorafenib-vorinostat dose matrix significantly reduced the viabilities in both ptCRC and mCRC PDOs with increasing concentrations of both drugs (Supplementary Fig. 7). In particular, this combination was more potent in CRC08 PDOs than the regorafenib-SN-38 combination was in CRC07 PDOs (Supplementary Fig. 7). However, the interactions between regorafenib and vorinostat also proved to be additive in nature via bliss synergy analysis in CRC08 PDOs (−10 < *δ* < 10) (Fig. 6e, f). Correspondingly, the leftward shift in the dose-response curve for the combination was only marginal in both PDOs (Fig. 6g, h). Nevertheless, phenotypic changes in CRC08 PDO morphology demonstrated that the QPOP-projected combination of regorafenib and vorinostat was still effective in mitigating metCRC PDO growth in vitro compared to their respective monotherapies (Supplementary Fig. 8b).

**Fig. 7:**
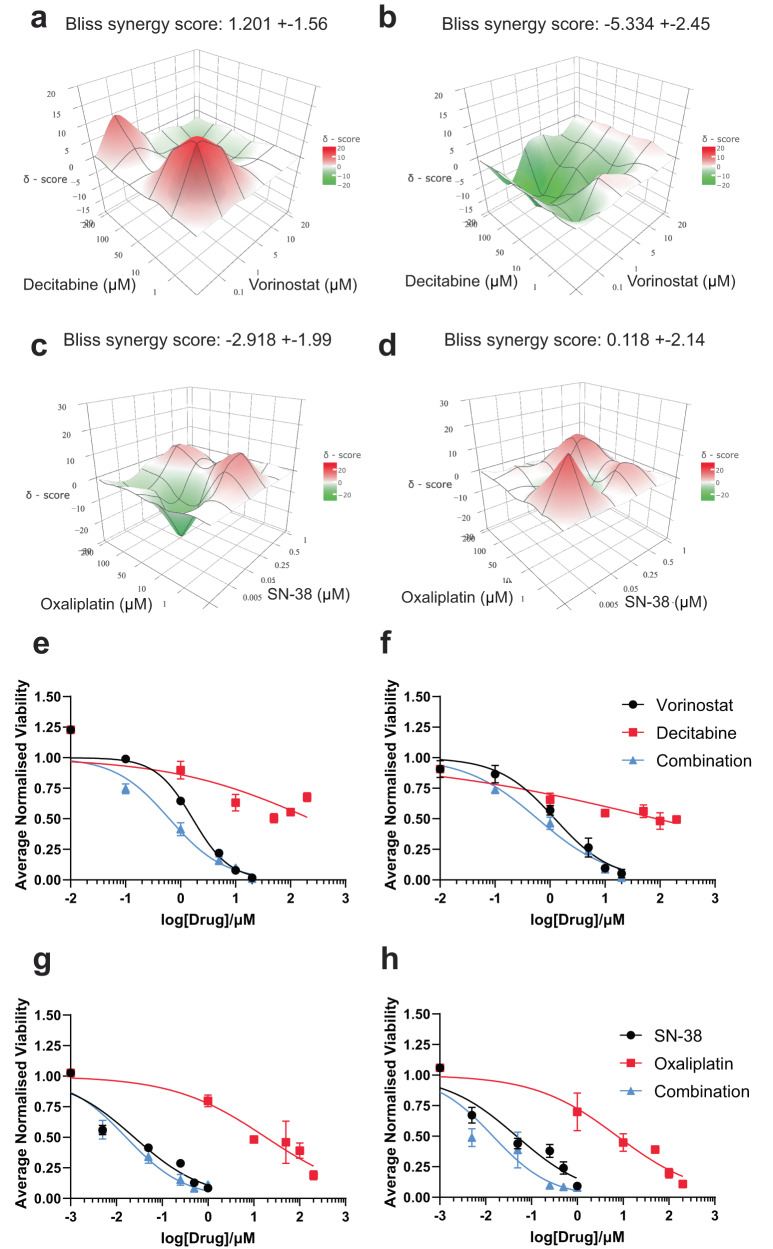
ptCRC11 and mCRC11 PDOs demonstrate different phenotypic response to QPOP-optimised combinations. **a** Bliss synergy maps for decitabine and vorinostat in ptCRC11 and **b** mCRC11 over the entire dose matrix. Bliss synergy scores are represented as means of the entire search space ± 95% confidence interval. **c** Synergy landscape for oxaliplatin-SN-38 in ptCRC11 and **d** mCRC11 over the entire dose matrix. Bliss synergy scores are represented as means of the entire search space ± 95% confidence interval. **e** Dose-response curves of decitabine and vorinostat in ptCRC11 and **f** mCRC11 organoids when administered singly and in combination. **g** Dose-response curves of ptCRC11 and **h** mCRC11 PDOs following oxaliplatin and SN-38 treatment alone and concurrently.

We next evaluated the efficacy of the two QPOP-optimised combination therapies for ptCRC11 and mCRC11 PDOs – decitabine-vorinostat and oxaliplatin-SN-38. Similar to CRC07 and CRC08, both combinations were effective in attenuating the viabilities of ptCRC11 and mCRC11 when treated with the drug dose matrix, especially at high concentrations (Supplementary Fig. 7). However, evaluation of the bliss synergy maps for both combinations demonstrated distinct differences in sensitivities of the matched PDOs, albeit still exhibiting additive interactions for both drug pairs (−10 < *δ* < 10) (Fig. 7a–d). Notably, decitabine and vorinostat exhibited greater synergy in ptCRC11 compared to mCRC11 (*δ* = 1.201 ± 1.56 and −5.334 ± 2.45, respectively) (Fig. 7a, b). Conversely, interactions between oxaliplatin and SN-38 were more antagonistic in ptCRC11 (*δ* = −2.918 ± 1.99 and 0.118 ± 2.14 respectively) compared to mCRC11 (Fig. 7c, d). Additionally, the dose-response curves for decitabine and vorinostat exhibited a greater shift in ptCRC11 whilst the curve for the oxaliplatin and SN-38 combination was markedly lower than its respective monotherapies in mCRC11 (Fig. 7e–h). Correspondingly, while both PDOs exhibited significant inhibition in organoid growth to both combinations at high concentrations, disruptions of the dense organotypic structures in ptCRC11 and mCRC11 were more distinct when treated with its QPOP-derived combinations at lower concentrations (Supplementary Fig. 9).

Collectively, the in vitro validation of the rationally optimised combinations in metCRC PDOs attested to the value of FPO in designing the best treatment strategy for metCRC patients presenting synchronous metastases as the drug sensitivity patterns of primary tumours and metastases exhibited significant inter-patient heterogeneity. While some matched tumours presented similar drug combination vulnerabilities, as in CRC07 and CRC08, a single combination systemic therapy would be insufficient in managing both the primary tumour and metastases for a subset of metCRC patients like CRC11. Instead, combinatorial treatment regimens for the latter cohort of patients would likely need to comprise of independently optimised systemic therapies to increase its coverage.

## Discussion

Current strategies for precision medicine are largely reliant on genomic characterisation of patient tumours. However, many frontline therapies for CRC patients, particularly chemotherapies, lack defined prognostic biomarkers given the inherently heterogeneous nature of the disease and diverse molecular profiles of patients^51–53^. FPO thus offers a promising and unbiased strategy at tailoring treatment strategies to patients based on the phenotypic response of the patients’ own tumour cells to pharmacological perturbations ex vivo. Growing efforts have thus been made towards the establishment of patient-derived models as clinically relevant disease models for numerous cancer types, including GI cancers, for this purpose^25,34,54–58^. Notably, the utilization of patient-derived avatars, especially organoids, have been gaining traction for CRC since the first living biobank of CRC PDOs was established in 2015^34^. To date, numerous groups have demonstrated that patient-derived models were able to successfully recapitulate the molecular profiles of metCRC, and are amenable for functional drug screening and investigations^25,34,35^. Importantly, the development of clinically relevant models for metCRC paved the way for the establishment of FPO pipelines with potential clinical applications. Here, we established our own cohort of 14 CRC PDOs from synchronous CRC primary tumours and metastases, including three pairs of matched metCRC PDOs, which preserved the histopathological characteristics and genomic aberrations specific to the corresponding parental tissues. Additionally, characterisation of the mutations in matched metCRC PDOs exhibited strong concordance with the clinical traits of the parental tumours^34,39,45–47^.

Consistent with existing literature, the metCRC PDOs also recapitulated the heterogeneous drug sensitivities of tumour cells to a panel of cancer therapeutics^24,34,35,59^. Further application of QPOP in matched metCRC PDOs highlighted their capabilities in screening drug combinations and modelling responses of tumour cells to drug combinations in vitro. Through downstream validation studies, the robustness of the metCRC PDOs as in vitro disease models for functional drug interrogation and the implementation of FPO was determined. More importantly, validation of in vitro organoid drug responses highlighted the significant inter-patient heterogeneity observed in terms of the pattern of PDO sensitivities to combination therapies at the individual level. Currently, there are few studies comparing the differential response of matched metCRC PDOs to drug combinations as compared to monotherapies, with only two recent studies demonstrating the varied responses of ptCRC and mCRC PDOs to frontline therapies for metCRC^24,60^. Concordantly, the three case studies presented in this study demonstrated the heterogeneous sensitivity patterns of matched metCRC PDOs to combination therapies. Notably, the results indicated that single-drug functional assays are insufficient in elucidating sensitivities of metCRC PDOs to combination therapies. Furthermore, the presence of patient-specific combinatorial vulnerabilities in metCRC patients highlights the necessity for personalised medicine in managing metCRC. However, a limitation of the drug studies performed in this study was that the confounding effects of the PDO growth rates on drug sensitivity were not accounted for^61^. Hence, it would be interesting to evaluate if the observations of this study would be recapitulated in larger cohorts of metCRC PDOs, which would expectedly exhibit greater inter-patient heterogeneity in the PDO growth rates, in downstream studies which factored in the growth rate of the cells.

Despite the differential patterns of response and resistance in the three pairs of matched metCRC PDOs in our study, mapping of the synergy between each drug pair was reflective of additive interactions and an absence of synergy in all QPOP-optimised drug combinations. This highlights a key limitation of using QPOP as a drug interrogation platform to identify optimal drug combinations from a panel of pre-selected drugs. Being an agnostic algorithm, QPOP models the interactions between drug pairs in a mechanism-blind manner^27,32^. Hence, if there were no inherent drug synergies within the panel of drugs, the optimised combination would not exhibit any synergy in the system and would be additive at best. Nevertheless, retrospective analysis of numerous clinical trials previously conducted highlighted that in vitro synergy is not necessary for a good indication of clinical efficacy^62^. Instead, the authors argued that independent drug action could elicit sufficient favourable patient response to warrant FDA approval for the management of cancer, attributable to the inter-patient heterogeneity of patient response to cancer therapeutics^62,63^. Taken together, the data suggests that the heterogeneous sensitivities of matched metCRC PDOs to QPOP-optimised combinations still present potentially clinically actionable regimens despite being only additive in nature, albeit requiring clinical validation. Additionally, the limited size of the drug panel is a limitation of QPOP as a FPO tool as the panel size may not be sufficiently large to represent all clinically relevant drugs. Moreover, QPOP does not consider the functional effects of sequentially administering the drugs as drug combinations were delivered to the PDOs concurrently.

Interestingly, unbiased design of optimal drug combinations for patient CRC08 and CRC11 revealed a general sensitivity towards epigenetic-based combinatorial vulnerabilities. The frequent inclusion of HDAC inhibitor vorinostat in combinations predicted to be more effective suggests that HDAC inhibition may be a key epigenetic vulnerability in metCRC tumours. Furthermore, prior studies have demonstrated a reliance on the modulation of histone acetylation in irinotecan-resistant CRC cells due to changes in the chromatin acetylation complex^17^. Functional drug assays performed in CRC08 PDOs corroborated the vulnerability of irinotecan-resistant cancer cells to the attenuation of HDAC activity, suggesting that HDAC inhibition could be a viable treatment strategy for metCRC patients who progressed on irinotecan-based combination therapies. Conceivably, vorinostat has thus been assessed in combination with 5-fluorouracil in clinical trials for metCRC patients following promising preclinical data^64–68^. However, while these combinations exhibited acceptable safety profiles, the efficacy was limited in clinical practice, suggesting that novel vorinostat-based therapies could be developed against metCRC^69,70^. Prior studies have reported findings which supported the combination of vorinostat with either tyrosine kinase inhibitors or DNMT inhibitors as optimised by QPOP analysis. In one study, the authors demonstrated that inhibition of the RAS oncogenic pathway could sensitise CRC cells to vorinostat, corroborating the identification of regorafenib-vorinostat as an effective combination against CRC08 PDOs^71^. The concurrent inhibition of DNMT and HDAC while less studied in CRC, has exhibited promising preliminary clinical efficacy in other solid cancer types, indicative of its therapeutic potential in patients^72–74^. Analyses performed on the functional response of metCRC PDOs therefore suggest that vorinostat-based drug combinations could be a viable treatment strategy for metCRC patients, albeit warranting further validation in a larger PDO and patient cohort.

Clinically, previous application of QPOP-guided FPO in lymphomas suggested that QPOP predictions of therapeutic sensitivity following ex vivo evaluation in primary patient cancer cells does translate to clinical concordance^31^. This has led to the subsequent conduct of clinical trials investigating the clinical prospects of QPOP as a tool for FPO in solid tumours, including breast cancer (NCT05177432), sarcoma (NCT04986748) and high grade astrocytic glioma (NCT05532397). Importantly, the necessity for established clinical concordance to support the FPO approach was evidenced in work presented previously, where the authors reported that their cohort of PDOs could not mimic the clinical response of patients to 5-fluorouracil and oxaliplatin in combination as it does for irinotecan-based combinations^35^. As QPOP-optimised systemic therapies are combinatorial in nature, it is therefore imperative that clinical validation studies be performed in the future, to ensure that the metCRC PDOs established can provide reliable drug responses that accurately recapitulate patient responses in the clinics.

Unfortunately, there is limited clinical data for the determination of concordance in patient and PDO sensitivity to the QPOP-derived drug combinations post-surgery as surgical resection was the primary treatment modality for patients recruited in this study, highlighting an important limitation of the study design. Instead, the FPO approach would be more relevant and essential in informing treatment strategies for patients later in the disease trajectory or in refractory patients who have progressed on multiple lines of frontline therapies. Matched PDOs from advanced metCRC patients deemed unsuitable for surgery could therefore be derived from biopsy-sampling in future clinical studies to establish the concordance between the functional response of patients and the matched metCRC PDOs to QPOP-designed combinations. However, biopsy sampling often yields lower cell counts, resulting in a lower PDO establishment rate^25,75^. Furthermore, dissection into the culture conditions which best support the generation of metCRC PDOs from different lesion sites is essential in improving the speed at which PDOs can be established for ex vivo drug sensitivity assays to be performed. To further the utilisation of metCRC PDOs in FPO for the management of the disease, it is therefore imperative to develop better technology which addresses the current limitations in organoid generation, improving the turnaround time which PDOs can be established and for drug sensitivity readouts to be made available in a clinically relevant timeframe^26^.

A final limitation of the current organoid system presented in this study for the progress of FPO is the inability of the current PDO system to mimic the tumour stromal heterogeneity^76^. Importantly, first-line systemic therapies for metCRC patients include anti-angiogenic bevacizumab and more recently, immune checkpoint inhibitors such as pembrolizumab and nivolumab^77–80^. Correspondingly, there is a need to develop metCRC PDO cultures in which tumours are co-cultured with exogenous components of the tumour microenvironment (TME), such as endothelial cells and immune cells to investigate the role of the TME in predicting patient response to first-line and emerging cancer therapies^81^.

More importantly, the work presented in here highlights the current limitations in personalised therapies guided by the functional response of PDOs established from a single lesion site. Instead, the heterogeneous landscape of patient sensitivity to drug combinations underscores the significance of applying FPO to multiple lesion sites, such as for patient CRC11. Differences in the response of ptCRC11 and mCRC11 to epigenetic targeted therapies, suggested that epigenomic variations between primary tumours and metastases may had contributed to the differential sensitivity profiles of primary and metastatic tumours. Correspondingly, a recent study identified distinctions in the DNA methylation patterns between primary tumours and liver metastases of metCRC patients, resulting in functional changes in the transcriptome of both lesions^21^. The results thus collectively demonstrate that the FPO approach, when applied concurrently to both primary tumours and metastases, adds a new dimension to precision and personalised oncology for metCRC, complementing current genomics-driven systemic treatment of metCRC.

In summary, the findings in this study have demonstrated the reliability of metCRC PDOs as disease models to guide treatment strategies for patients using a FPO approach, through the conduct of ex vivo functional drugs screens. Leveraging on QPOP as a rational drug combination optimisation tool, we demonstrated the presence of differential combinatorial vulnerabilities in both ptCRC and mCRC PDOs which could be exploited for disease treatment. Interestingly, the varied inter-patient response of the metCRC PDOs to epigenetic drug combinations underscores the potential of personalising epigenetic therapies for metCRC patients. Our findings therefore collectively highlight the promise of the FPO approach in recommending the best combination of systemic treatments for metCRC patients with synchronous primary tumours and metastases, potentially changing the landscape of metCRC management in the future.

## Methods

### Establishment of patient-derived organoid cultures

Matched CRC patient tumours from primary tumour sites and corresponding metastases were received from National University Hospital (NUH) for the establishment of PDOs with study approval from the NUH-domain-specific review board (DSRB) (NHG-DSRB Ref: 2019/00817) and National University of Singapore (NUS)-Institutional Review Board (IRB) (NUS-IRB Ref: LH-18-032E). All samples were collected with written informed consent from patients and in compliance with all relevant ethical regulations, including the Declaration of Helsinki.

Tumour organoids were established from CRC patient material as described previously^32,34,82,83^. Briefly, patient tumours were briefly minced and incubated with 1 mg/ml collagenase/dispase® (Roche) for 30 min before being passed through a 100 µM cell strainer. Following centrifugation, tumour cells were treated with 1× red blood cell lysis buffer (Invitrogen) on ice for 3 min and 1 mg/ml DNase I (Roche) (as necessary). Following subsequent centrifugation, isolated tumour cells were seeded in 80% Matrigel™ (v/v) and cultured in DMEM/F-12 (Biowest) supplemented with growth factors necessary for respective CRC cultures as described previously^34,84^.

Organoid medium was changed and replenished twice a week, and CRC PDOs were passaged every 14 to 28 days as described previously^82^. Embedded organoids were incubated with 50% Cultrex™ organoid harvesting solution (R&D Systems) for an hour to release the organoids before centrifugation at 300 g at 4 °C. Isolated organoids were digested with either StemPro™ Accutase™ (Gibco) or TrypLE™ Express (Gibco) (where appropriate) for 3 min and treated with 1 mg/ml DNase I (Roche). Digested organoids were then seeded at a density of 5000 organoids per well for culture.

### Immunohistochemistry

Primary tissues from CRC patients and PDOs were fixed in 10% neutral buffered formalin (Sigma) overnight and embedded in paraffin before tissue sectioning. Tissue sections were deparaffinised and rehydrated for subsequent staining. Serial staining with haematoxylin and eosin (H&E) (Leica Biosystems) was performed for histological analysis of CRC primary tissues and PDOs. CRC primary tissues were also stained with antibodies for common markers of CRC (Supplementary Table 6). Briefly, rehydrated tissue sections were subject to heat mediated antigen retrieval with Target Retrieval Solution, pH 6.0 (Dako) followed by serial incubation in peroxidase-blocking solution (Dako) and antibody diluent (Perkin Elmer). The slides were then incubated with primary antibodies overnight, and the EnVision Detection Kit (Dako) used to generate the signal from the diaminobenzidine-HRP catalytic reaction. Antibody-stained tissue sections were then counterstained with haematoxylin. All stained tissue and PDO sections were subsequently dehydrated and mounted with CV Ultra mounting media (Leica Biosystems). Image acquisition of stained sections was performed using the AxioPlan 2 Imaging microscope (Zeiss).

### Immunofluorescence

Organoids were seeded in black 96 well cell culture microplates (Greiner)for a week and fixed in 4% paraformaldehyde (Sigma) overnight before permeabilization with 0.5% Triton X-100 (Sigma) for an hour. Organoids were subsequently incubated in blocking buffer (10% normal horse serum (Biowest), 0.1% bovine serum albumin (Biowest) and 0.2% triton X-100) overnight, followed by respective primary antibodies overnight. Lastly, organoids were incubated in secondary antibodies conjugated with either Alexa Fluor® 488 (Invitrogen) or Alexa Fluor® 594 (Life Technologies) and stained with DAPI (ThermoFisher Scientific) overnight before they were washed. Imaging of CRC organoids were performed using the EVOS M700 Imaging System (ThermoFisher Scientific). Details of antibodies used are listed in Supplementary Table 6.

### Whole exome sequencing and mutational profiling

Genomic DNA (gDNA) from primary CRC tissues and CRC PDOs were isolated using the DNeasy Blood & Tissue Kit (Qiagen) in accordance with the manufacturer’s instructions. Whole exome sequencing of extracted gDNA was performed by NovogeneAIT Genomics Singapore. Briefly, sample quantification and integrity were assessed using the Qubit® DNA Assay Kit in Qubit® 2.0 Flurometer (Invitrogen) and via agarose gel electrophoresis analysis respectively prior to sequencing. gDNA samples were then sheared into short fragments of 180–280 base pairs and subjected to whole exome sequencing using the SureSelect Human All Exon V6 capture probe set (Agilent) and the NovaSeq 6000 (Illumina) with PE150 strategy using 150 base pairs paired-end reads.

Variant calling was performed in accordance with Genome Analysis ToolKit (GATK) best practice. Briefly, paired-end clean reads from sequencing results were aligned against the human reference genome GRCh38 using the Burrows-Wheeler Aligner (v0.7.17-r1188)^85^. Variant calling was performed using GATK (v4.2.5) MuTect2 following removal of duplicated reads and base quality recalibration using the Picard suite (v2.28.0)^86^. Variants were additionally called using muTect (v1.1.4) and Strelka (v2.9.4) (muTect-Strelka)^87,88^. Variants were subsequently annotated using ANNOVAR (v2020-06-07)^89^. Variants annotated as benign/likely benign in ClinVar (v20220320) with population allele frequency > 0.01 in ExAC03 and gnomAD were excluded from analyses^90^. Variants that were identified by both MuTect2 and muTect-Strelka, or pathogenic germline mutations reported in colorectal cancer were retained for analyses. Phylogenetic trees were plot based on the shared and non-shared mutations present in three pairs of metCRC primary tumours and metastases, as well as matched PDOs using R (v4.2.2) MesKit package (v1.10.0)^91^. Maximum parsimony was used to construct the corresponding phylogenetic trees.

### Determination of half-maximal inhibitory concentrations (IC_50_)

Organoids were seeded in white 384 well microplates (NEST Biotech) and subsequently pre-treated with log dose concentrations of various pharmacological agents for 120 hours. CRC PDOs were treated with 10 drugs, namely standard of care chemotherapies 5-fluorouracil (Selleckchem), oxaliplatin (Selleckchem), leucovorin (Selleckchem), SN-38 (active metabolite of irinotecan) (Selleckchem), multikinase inhibitor, regorafenib (Selleckchem), PRMT5 inhibitor, pemrametostat (Selleckchem), PRMT4 inhibitor, TP-064 (Tocris), DNA methyltransferase inhibitor, decitabine (Selleckchem), and histone deacetylase inhibitors, entinostat (Selleckchem) and vorinostat (Selleckchem). Organoid viability was determined via CellTiter-Glo® luminescent assays (Promega). Sigmoidal dose-response curves were then generated using Prism 9 software (GraphPad) from which the respective half-maximal inhibitory concentrations (IC_50_) and area under the curves (AUCs) were interpolated.

### Quadratic phenotypic optimization platform (QPOP) combinatorial drug treatment and analyses

CRC PDOs were screened against drug combination iterations derived from an orthogonal array composite design (OACD) to streamline the drug combination interrogation pipeline as developed previously^50^. The OACD combines a two-level fractional factorial design and a three-level orthogonal array to generate the least number of combinations necessary for sufficient screening of factors and in-depth analyses^50^. CRC PDOs were screened with drug combinations derived from OACD using a resolution IV factorial design for 10 drugs, giving rise to a total of 91 combinations (Supplementary Table 3). The respective drug combinations for each OACD iteration were prepared at the respective IC_15_ and IC_30_ concentrations of each drug derived from the sigmoidal dose-response curves generated. In the event the IC_50_ for the drug exceeds the corresponding maximum serum concentration (*C*_max_) of the drug, 10% and 20% *C*_max_ concentrations will be used instead to ensure greater clinical relevance of the QPOP analyses. Drug combinations were prepared using the JANUS® G3 automated liquid handler (Perkin Elmer) and screened against CRC PDOs 24 h after organoids seeding. Organoid viability of CRC PDOs were determined 120 and 48 hours post-drug combination treatment respectively using CellTiter-Glo® luminescent assay.

Viabilities of organoids were used as input data for QPOP analyses as established previously^27^. Briefly, the phenotypic viabilities were fitted into a second-order quadratic series using Optim.AI™ (Kyan Technologies). The generated regression model was then used to generate the projected PDO viabilities for all possible combinations within the search space. The geometric mean of all two-drug combinations were subsequently computed and stratified into percentiles for the generation of the polygonograms. Parabolic surface responses were also generated based on the QPOP regression analysis to depict the interactions between each drug pair.

### Validation of QPOP-derived drug combinations

Paired metCRC PDOs were treated with log dose concentration matrix of the respective QPOP-optimised drug pairs for 120 h before organoid viability was measured using CellTiter-Glo® luminescent assay. Organoid viabilities were used to determine the bliss synergy score for the respective drug pairs. Bliss synergy maps were additionally generated to illustrate the synergy landscape across the entire dose matrix. Bliss synergy analysis was performed on the SynergyFinder+ web application^92^. Brightfield images of CRC organoids were obtained using the EVOS M700 Imaging System.

### Statistical analysis

All experiments were performed in triplicates, unless otherwise stated. All data are presented as means ± standard deviation. Two-way ANOVA was used to determine the statistical difference within groups when more than two variables were involved, and correction for multiple pairwise comparisons was performed according to the software’s recommendations. For QPOP analyses, sum of squares *F*-test was performed for each parameter estimation and the adjusted *R*^2^ value was used to determine the fidelity and robustness of the QPOP-generated model. All statistical analyses were performed on the Prism 9 software (GraphPad), while all analyses for QPOP were performed on Optim.AI™ (Kyan Technologies). Statistical significance was set at a threshold of *p*-value less than 0.05 unless otherwise stated.

### Reporting summary

Further information on research design is available in the Nature Research Reporting Summary linked to this article.

### Supplementary information

Supplementary Information

REPORTING SUMMARY

## Data Availability

The phenotypic drug sensitivity data supporting the findings of this study are available upon reasonable request from the corresponding author. However, the paired patient genomic data cannot be publicly shared due to the ethical considerations from the NUH-domain-specific review board and are available from the corresponding author upon reasonable request through collaborative investigations. The paired PDO genomic data have been deposited on the Sequence Read Archive (SRA) repository (BioProject accession number PRJNA1068196).

## Code availability

Detailed code for the QPOP analyses is unavailable as the software, Optim.AI™, is proprietary to Kyan Technologies.
