# Supplementary material for: A functional personalised oncology approach against metastatic colorectal cancer in matched patient derived organoids
Source: NPJ Precis Oncol. 2024 Feb 27;8:52. doi: 10.1038/s41698-024-00543-8 (PMC10899621; doi:10.1038/s41698-024-00543-8)
Supplement: Supplementary file 1 — Supplementary Information [file 41698_2024_543_MOESM1_ESM.pdf]

Supplementary Figures

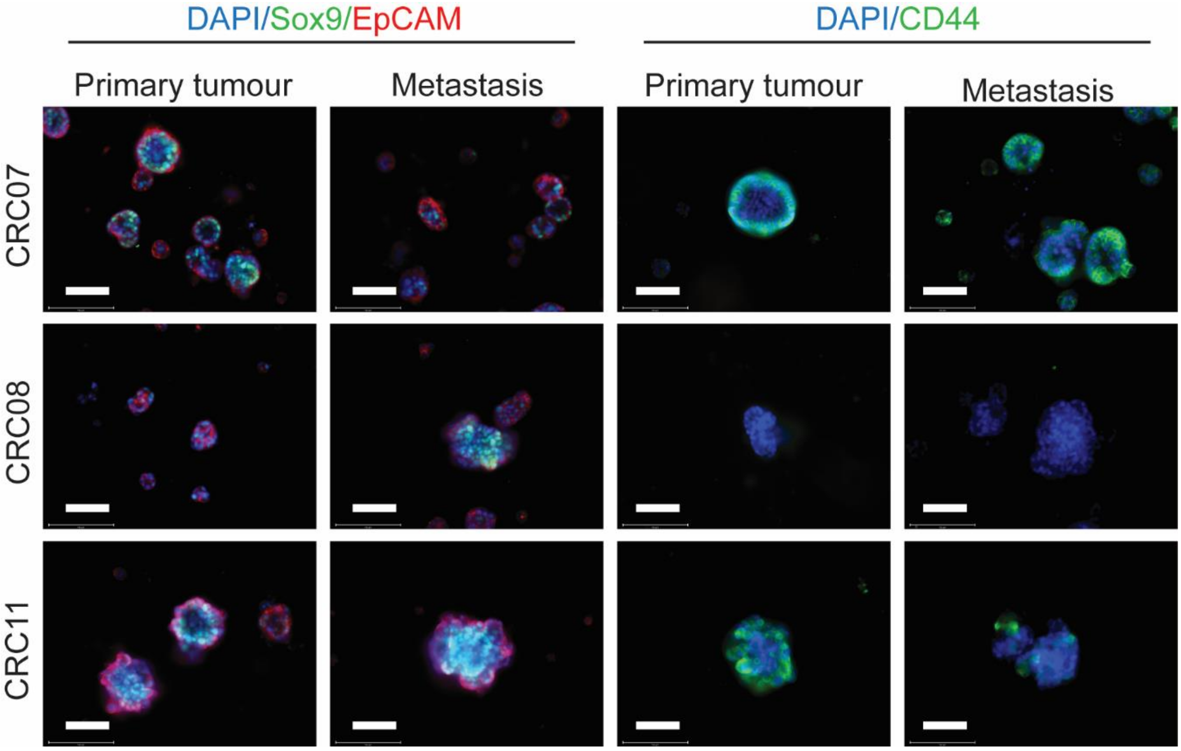

Supplementary Figure 1. Characterisation of cancer stem cell markers in matched metCRC PDOs. Immunofluorescence of cancer stem cell markers, Sox9, EpCAM and CD44, in paired primary tumour- and metastasis-derived CRC PDOs. Scale bar = 100 µm.

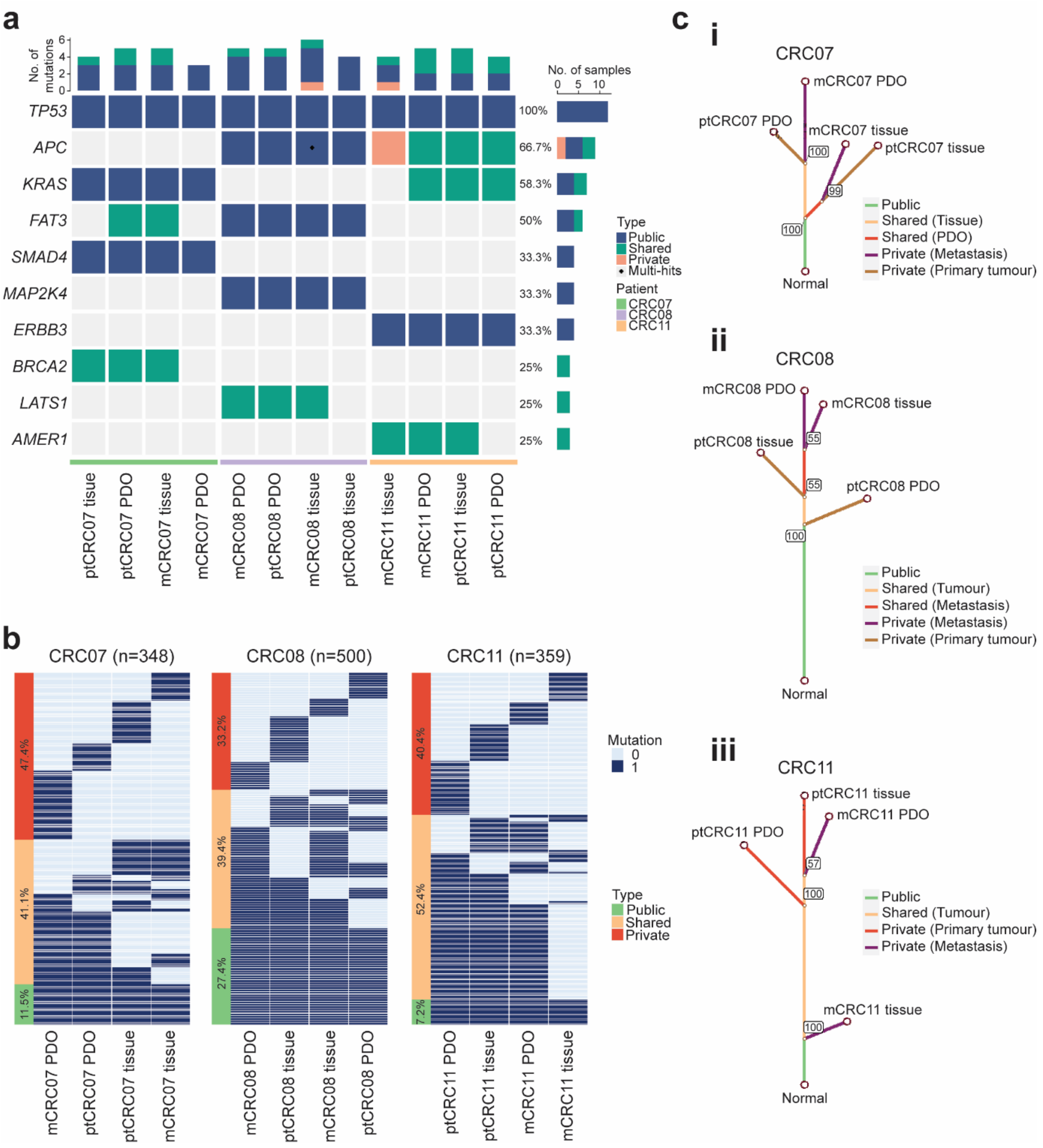

Supplementary Figure 2. Phylogenetic analysis of metCRC PDOs in culture. (a) Phylogenetic profile of mutations in common CRC driver genes identified in our cohort. **(b)** Heatmap of shared and non-shared mutations identified in the samples derived from three metCRC patients. **(c)** Phylogenetic trees for **(i)** CRC07, **(ii)** CRC08 and **(iii)** CRC11 based on the overlap in shared and non-shared mutations depicted in **(B)**. The number at each node corresponds to the number of acquired mutations in the branch population.

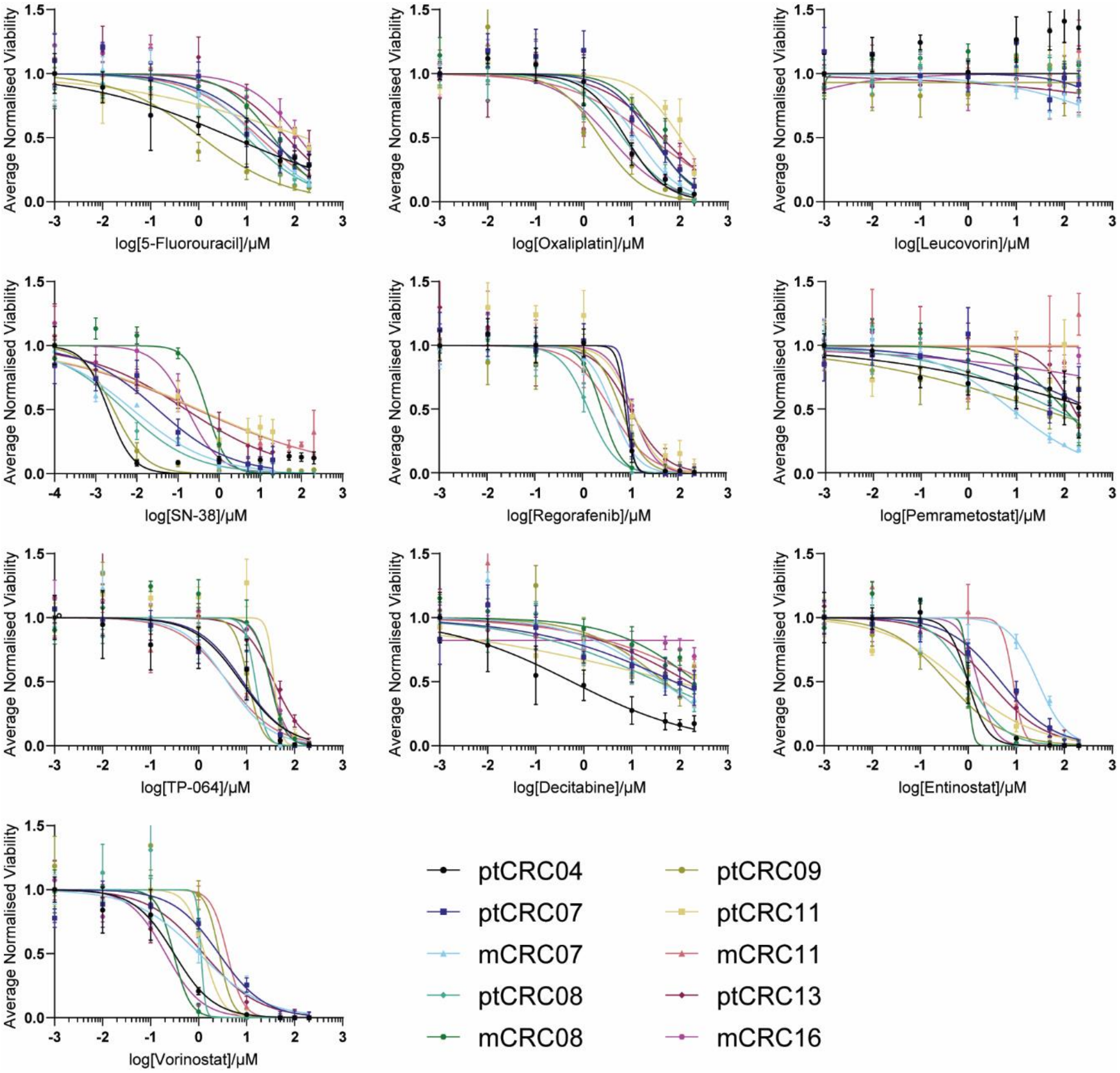

Supplementary Figure 3. CRC PDOs are amenable to drug screening. Representative dose-response curves for the 10 cancer therapeutics in 10 PDO lines (n = 3).

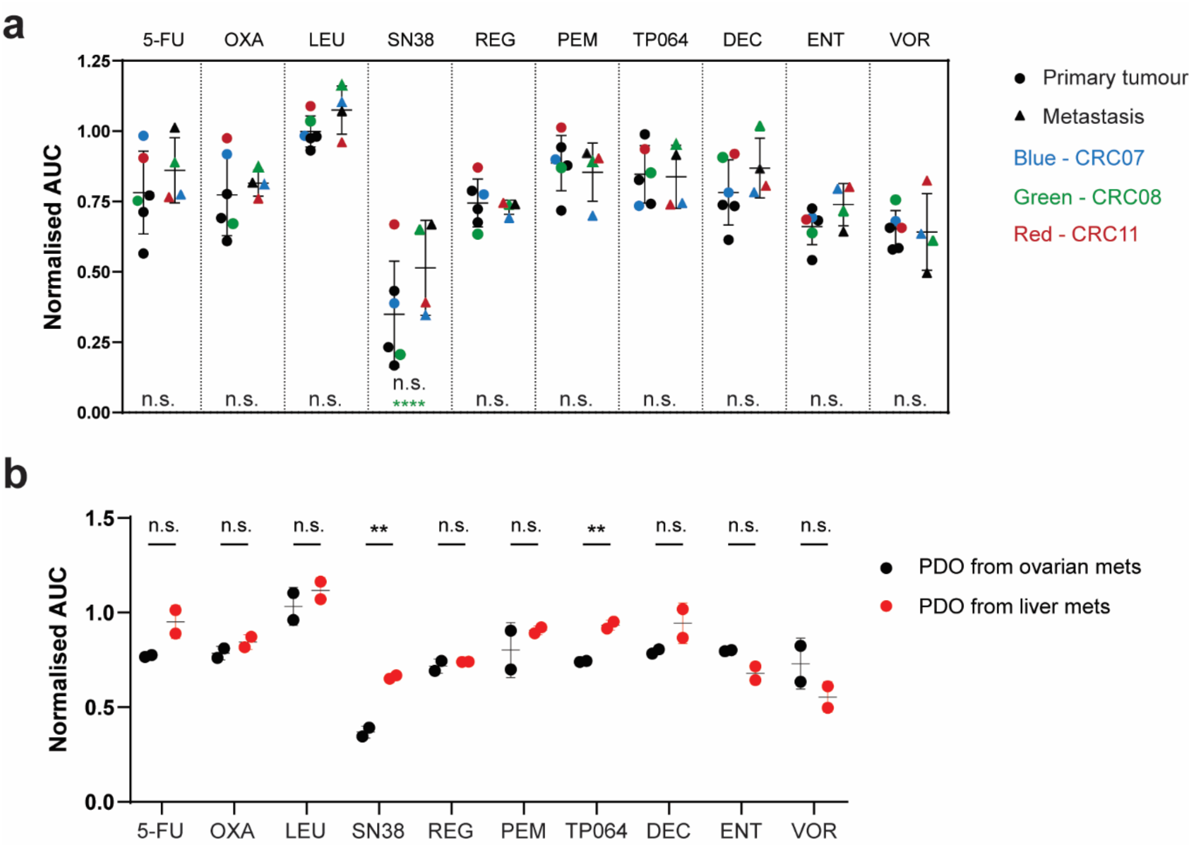

Supplementary Figure 4. metCRC PDOs demonstrate similar drug responses. (a) Summary of normalised AUC values when PDOs were grouped according to tumour type. Matched PDOs were highlighted in blue (CRC07), green (CRC08) and red (CRC11). Two-way ANOVA and Šidák's pairwise comparisons were performed as recommended (n.s.: not significant; ****p < 0.0001). Data represented as means ± SD (n = 6 and 4 for primary tumour and metastasis PDOs respectively). **(b)** Overview of normalised AUC when metastases PDOs were stratified based on metastatic site. Two-way ANOVA and Šidák's pairwise comparisons were performed as recommended (n.s.: not significant; **p < 0.01). Data represented as means ± SD (n = 2). (5-FU, 5-fluorouracil; OXA, oxaliplatin; LEU, leucovorin; SN38, SN-38; REG, regorafenib; PEM, pemrametostat; TP064, TP-064; DEC, decitabine; ENT, entinostat; VOR, vorinostat)

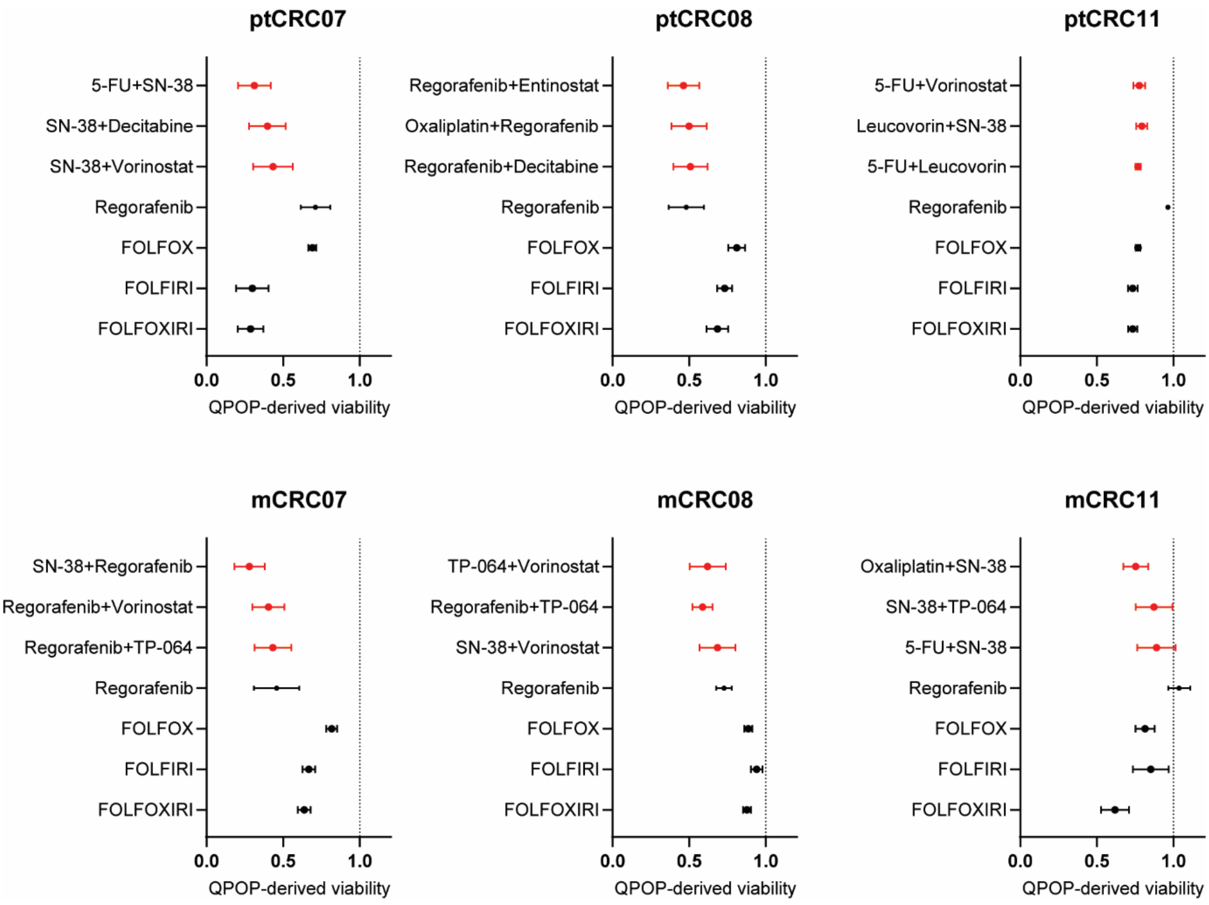

Supplementary Figure 5. QPOP identified tumour-specific vulnerabilities in metCRC PDOs. Forest plots of the expected viabilities of metCRC PDOs in response to the top-ranked two-drug combinations (in red) and standard systemic regimens, which include three- and four-drug combinations (FOLFOX, FOLFIRI and FOLFOXIRI in black), based on the QPOP analysis. Data represented as means ± SD from QPOP analysis (n = 2 for single-drug therapy regorafenib, n = 4 for top-ranking two-drugs combinations, n = 8 for three-drugs combinations FOLFOX and FOLFIRI, n = 16 for four-drugs combination FOLFOXIRI). (FOLFOX, 5-fluorouracil + leucovorin + oxaliplatin; FOLFIRI, 5-fluorouracil + leucovorin + irinotecan [SN-38]; FOLFOXIRI, 5-fluorouracil + leucovorin + oxaliplatin + irinotecan [SN-38])

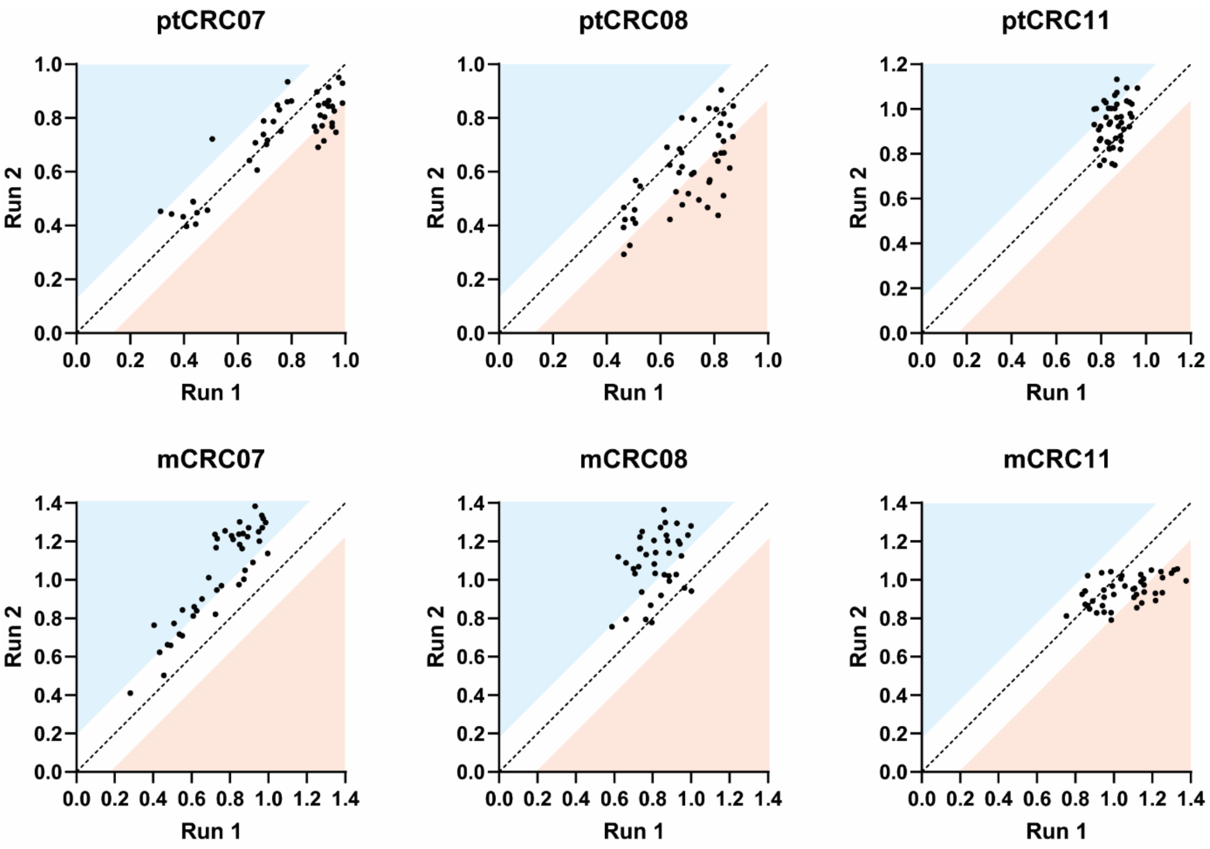

Supplementary Figure 6. QPOP results are largely consistent between biological replicates. Correlation between the QPOP-generated organoid viabilities of doublet drug combinations in three pairs of matched ptCRC and mCRC PDOs from two biological replicates. Data points in the blue triangles are indicative of drug combinations with greater efficacy in the first QPOP analysis. Data points in the red triangles are reflective of doublet combinations inducing greater cell death in the second QPOP experiment. Majority of the data points are reflective of the consistency between two iterations of QPOP analysis.

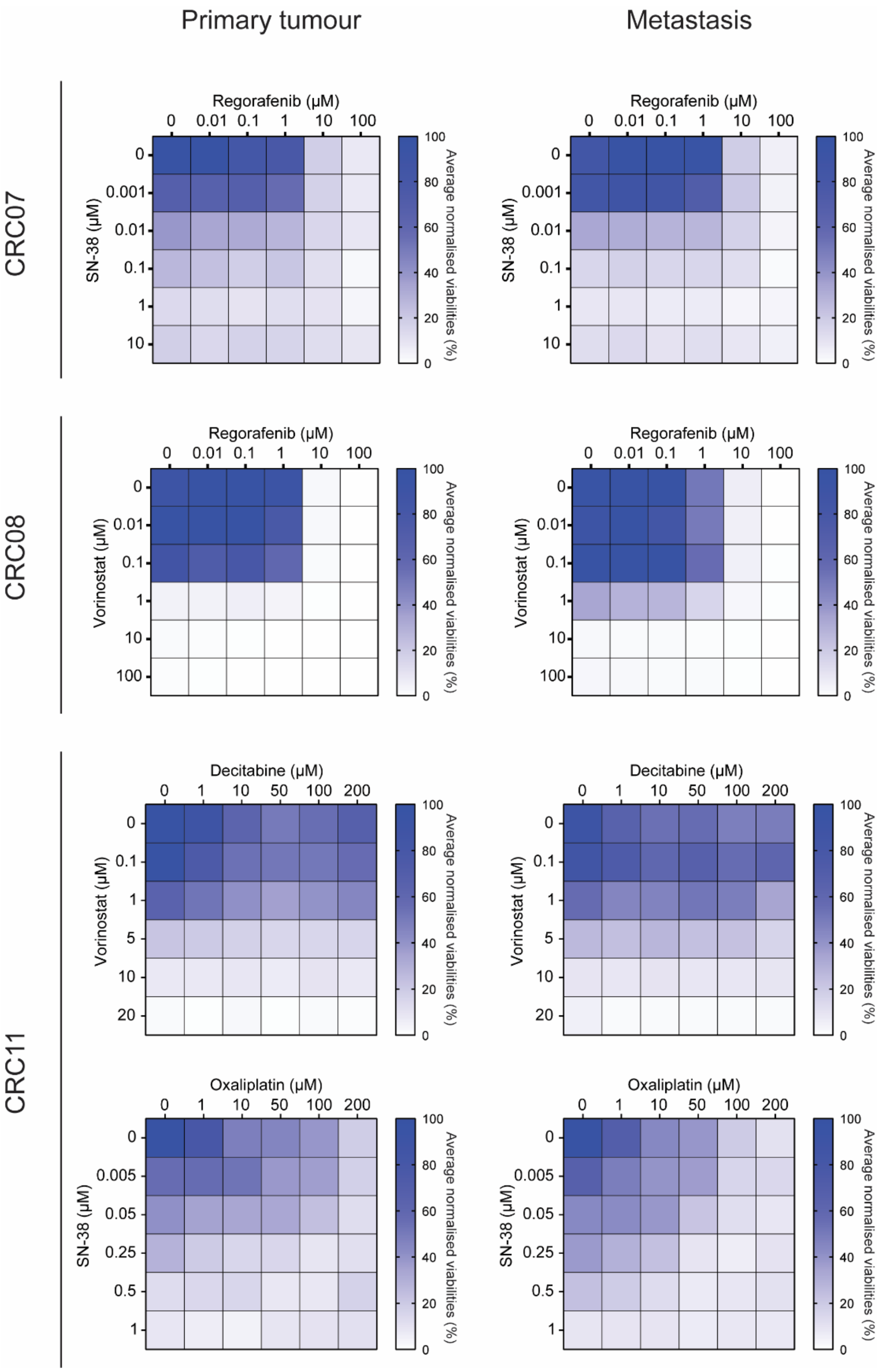

Supplementary Figure 7. CRC PDOs exhibit heterogeneous patterns in response to drug combinations. Organoid viabilities of paired ptCRC and mCRC PDOs in response to dose matrix of its respective QPOP-optimised drug combinations (n = 2).

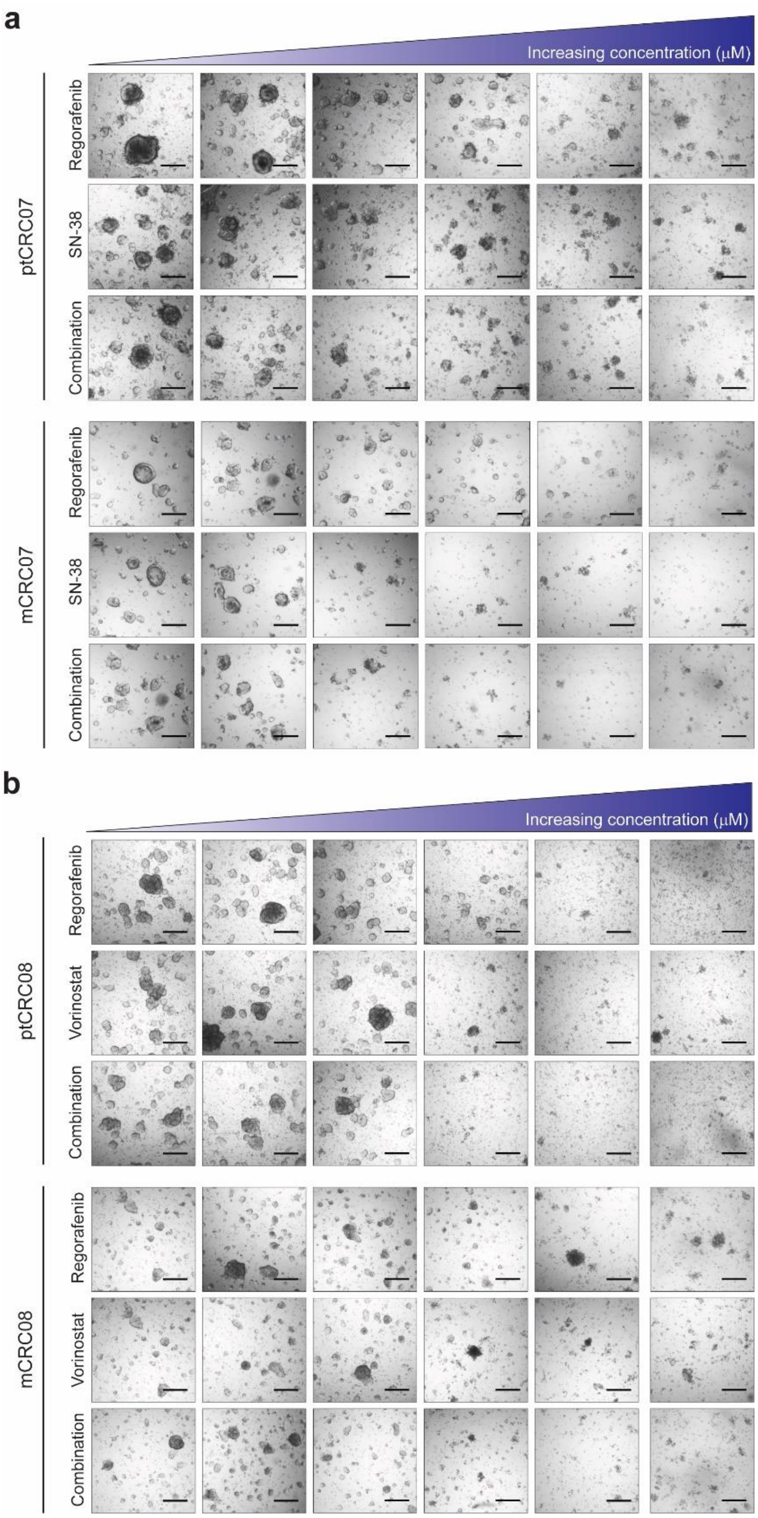

Supplementary Figure 8. CRC07 and CRC08 PDOs exhibit similar responses to QPOP-optimised drug combinations. (a) Corresponding brightfield images of ptCRC07 and mCRC07 PDOs when treated with increasing dosages of regorafenib and SN-38. Scale bar = 250 µm. **(b)** Corresponding brightfield organoid pictures of ptCRC08 and mCRC08 when treated with increasing concentrations of regorafenib and vorinostat. Scale bar = 250 µm.

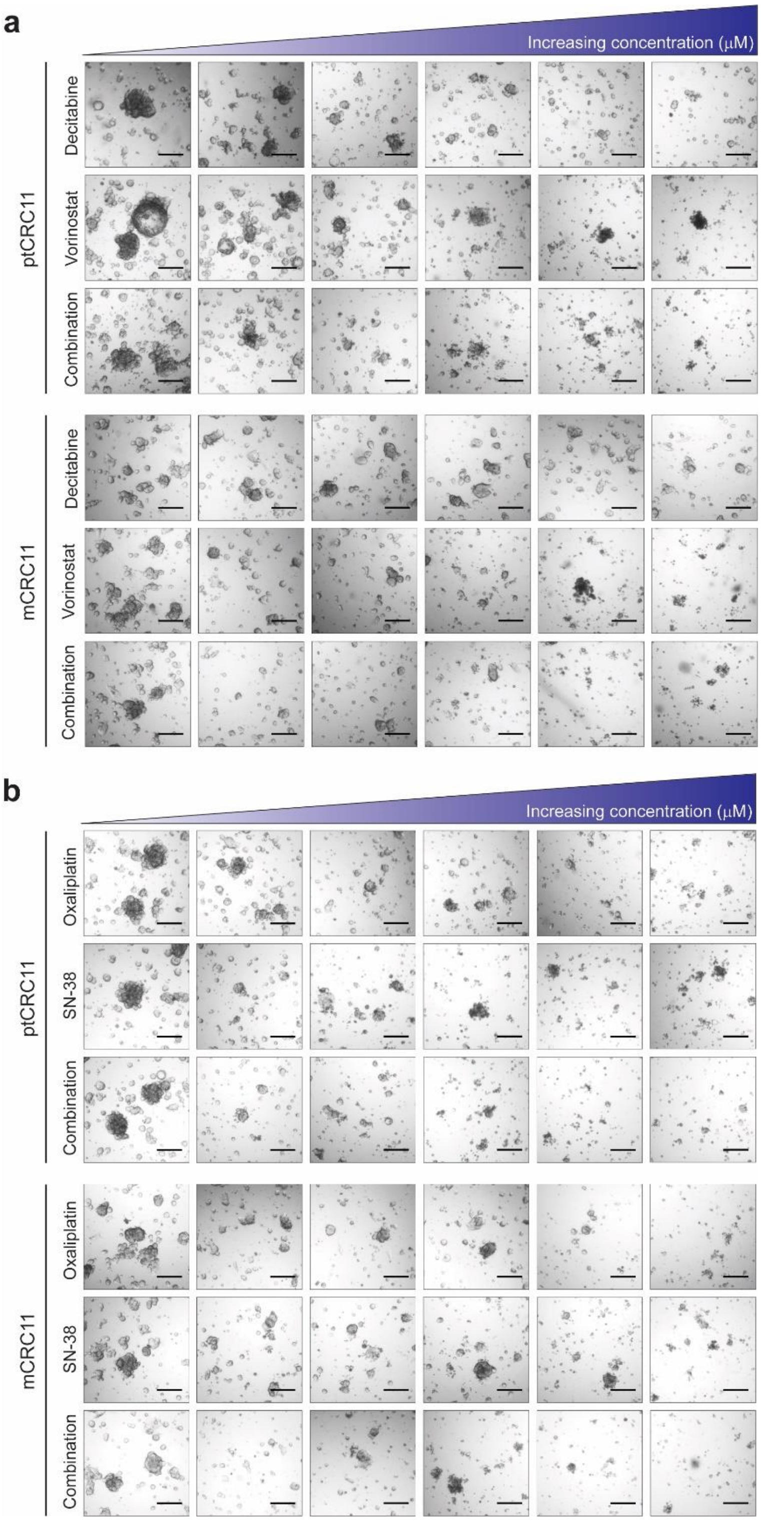

Supplementary Figure 9. ptCRC11 and mCRC11 PDOs demonstrate different phenotypic response to QPOP-optimised combinations. (a) Corresponding brightfield organoid images of ptCRC11 and mCRC11 when treated with increasing dosages of decitabine and vorinostat. Scale bar = 250 µm. **(b)** Brightfield images of ptCRC11 and mCRC11 PDOs when treated with increasing dosages of oxaliplatin and SN-38. Scale bar = 250 µm.

Supplementary Tables

Supplementary Table 1. Demographic and clinicopathological characteristics of 17 colorectal patients enrolled in this study and corresponding organoids established.

Sample code	Metastatic / Non-metastatic	Primary lesion				Metastatic Site				Clinical characteristics						
		Location of Primary lesion	Primary tissue collected (Yes/No)	Viable cells (Yes/No)	ptCRC organoid status	Location of metastatic lesion	Metastatic tissue collected (Yes/No)	Viable cells (Yes/No)	mCRC organoid status	Age	Sex (F/M)	Histological type	Grade of differentiation	Pathological T stage	Pathological N stage	Pathological M stage
CRC01	Metastatic	Unspecified	No	NA	NA	Liver	Yes	Yes	Fungal contamination	51	M	Unspecified	Unspecified	Unspecified	Unspecified	Unspecified
CRC02	Metastatic	Sigmoid	Yes	Yes	Fungal contamination	Liver	Yes	Yes	Fungal contamination	56	M	Adenocarcinoma	Moderate	T3	N1	M1
CRC04	Metastatic	Sigmoid	Yes	Yes	Success (ptCRC04)	Liver	Yes	No	NA	54	M	Adenocarcinoma	Well	T2	N2	M1
CRC05	Metastatic	Sigmoid	Yes	No	NA	Liver	Yes	No	NA	53	F	Adenocarcinoma	Unknown	Unknown	N0	Unknown
CRC07	Metastatic	Cecum	Yes	Yes	Success (ptCRC07)	Ovaries	Yes	Yes	Success (mCRC07)	41	F	Adenocarcinoma	Well to moderate	T4	N2	M1
CRC08	Metastatic	Rectum	Yes	Yes	Success (ptCRC08)	Liver	Yes	Yes	Success (mCRC08)	47	F	Adenocarcinoma	Poor	T3	N1	M1
CRC09	Metastatic	Descending	Yes	Yes	Success (ptCRC09)	Uterine Myometrium	No	NA	NA	36	F	Adenocarcinoma	Well to moderate	T4	N2	M1
CRC11	Metastatic	Cecum	Yes	Yes	Success (ptCRC11)	Ovaries	Yes	Yes	Success (mCRC11)	73	F	Adenocarcinoma	Moderate	T4	N2	M1
CRC12	Metastatic	Cecum and Ascending	Yes	Yes	Success (ptCRC12)	Liver	No	NA	NA	66	F	Adenocarcinoma	Well to moderate	T4	N1	M1
CRC13	Metastatic	Sigmoid	Yes	Yes	Success (ptCRC13)	Liver	No	NA	NA	76	M	Adenocarcinoma	Poor	T3	N1	M1
CRC15	Non-metastatic	Rectum	Yes	Yes	Success (ptCRC15)	NA	NA	NA	NA	65	F	Adenocarcinoma	Well to moderate	T3	N0	M0
CRC16	Metastatic	Sigmoid	No	NA	NA	Liver	Yes	Yes	Success (mCRC16)	55	M	Adenocarcinoma	Well to moderate	T4	N1	M1
CRC17	Metastatic	Sigmoid	Yes	Yes	Success (ptCRC17)	Liver	Yes	Yes	Failure	54	M	Adenocarcinoma	Moderate	T3	N0	M1
CRC19	Non-metastatic	Rectosigmoid	Yes	No	NA	NA	NA	NA	NA	68	M	Adenocarcinoma	Well to moderate	T4	N0	M0
CRC20	Non-metastatic	Splenic Flexure	Yes	Yes	Failure	NA	NA	NA	NA	48	M	Adenocarcinoma	Well to moderate	T3	N1	M0
CRC21	Non-metastatic	Ascending Colon	Yes	Yes	Failure	NA	NA	NA	NA	66	F	Adenocarcinoma	Well to moderate	T4	N0	M0
CRC22	Non-metastatic	Sigmoid	Yes	Yes	Success (ptCRC22)	NA	NA	NA	NA	79	F	Adenocarcinoma	Moderate	T3	N0	M0

Supplementary Table 2. Pathways frequently mutated in matched metCRC parental tissues and PDOs.

Pathway	Number of genes in the pathway	Number of affected genes	Fraction of genes affected	Number of mutated samples	Fraction of samples mutated	Affected genes
Cell Cycle	15	1	0.06666667	1	0.08333333	*CDK2*
TP53	6	1	0.16666667	12	1	*TP53*
MYC	13	2	0.15384615	3	0.25	*MXD4, MNT*
TGF-Beta	7	2	0.28571429	5	0.41666667	*SMAD4, TGFBR1*
NOTCH	71	3	0.04225352	4	0.33333333	*LFNG, NCOR2, SPEN*
RTK-RAS	85	4	0.04705882	12	1	*KRAS, ERBB3, KSR1, SHC2*
WNT	68	7	0.10294118	10	0.83333333	*APC, FZD10, AMER1, WNT4, LZTR1, TCF7L2, WNT7A*
Hippo	38	7	0.18421053	10	0.83333333	*FAT3, DCHS1, LATS1, CRB1, FAT2, FAT4, FAT1*

Supplementary Table 3. QPOP combination design using orthogonal array composite design consisting of 91 combinations for 10 drugs at three increment dosages (-1, 0, 1).

Combination	Drug 1	Drug 2	Drug 3	Drug 4	Drug 5	Drug 6	Drug 7	Drug 8	Drug 9	Drug 10
1	-1	-1	-1	-1	-1	-1	-1	1	-1	1
2	-1	-1	-1	-1	-1	1	-1	-1	1	-1
3	-1	-1	-1	-1	1	-1	1	1	-1	-1
4	-1	-1	-1	-1	1	1	1	-1	1	1
5	-1	-1	-1	1	-1	-1	1	1	1	1
6	-1	-1	-1	1	-1	1	1	-1	-1	-1
7	-1	-1	-1	1	1	-1	-1	1	1	-1
8	-1	-1	-1	1	1	1	-1	-1	-1	1
9	-1	-1	1	-1	-1	-1	1	-1	-1	1
10	-1	-1	1	-1	-1	1	1	1	1	-1
11	-1	-1	1	-1	1	-1	-1	-1	-1	-1
12	-1	-1	1	-1	1	1	-1	1	1	1
13	-1	-1	1	1	-1	-1	-1	-1	1	1
14	-1	-1	1	1	-1	1	-1	1	-1	-1
15	-1	-1	1	1	1	-1	1	-1	1	-1
16	-1	-1	1	1	1	1	1	1	-1	1
17	-1	1	-1	-1	-1	-1	1	-1	-1	-1
18	-1	1	-1	-1	-1	1	1	1	1	1
19	-1	1	-1	-1	1	-1	-1	-1	-1	1
20	-1	1	-1	-1	1	1	-1	1	1	-1
21	-1	1	-1	1	-1	-1	-1	-1	1	-1
22	-1	1	-1	1	-1	1	-1	1	-1	1
23	-1	1	-1	1	1	-1	1	-1	1	1
24	-1	1	-1	1	1	1	1	1	-1	-1
25	-1	1	1	-1	-1	-1	-1	1	-1	-1
26	-1	1	1	-1	-1	1	-1	-1	1	1
27	-1	1	1	-1	1	-1	1	1	-1	1
28	-1	1	1	-1	1	1	1	-1	1	-1
29	-1	1	1	1	-1	-1	1	1	1	-1
30	-1	1	1	1	-1	1	1	-1	-1	1
31	-1	1	1	1	1	-1	-1	1	1	1
32	-1	1	1	1	1	1	-1	-1	-1	-1
33	1	-1	-1	-1	-1	-1	1	-1	1	-1
34	1	-1	-1	-1	-1	1	1	1	-1	1
35	1	-1	-1	-1	1	-1	-1	-1	1	1
36	1	-1	-1	-1	1	1	-1	1	-1	-1
37	1	-1	-1	1	-1	-1	-1	-1	-1	-1
38	1	-1	-1	1	-1	1	-1	1	1	1
39	1	-1	-1	1	1	-1	1	-1	-1	1
40	1	-1	-1	1	1	1	1	1	1	-1
41	1	-1	1	-1	-1	-1	-1	1	1	-1
42	1	-1	1	-1	-1	1	-1	-1	-1	1
43	1	-1	1	-1	1	-1	1	1	1	1
44	1	-1	1	-1	1	1	1	-1	-1	-1
45	1	-1	1	1	-1	-1	1	1	-1	-1
46	1	-1	1	1	-1	1	1	-1	1	1
47	1	-1	1	1	1	-1	-1	1	-1	1
48	1	-1	1	1	1	1	-1	-1	1	-1
49	1	1	-1	-1	-1	-1	-1	1	1	1
50	1	1	-1	-1	-1	1	-1	-1	-1	-1

51	1	1	-1	-1	1	-1	1	1	1	-1
52	1	1	-1	-1	1	1	1	-1	-1	1
53	1	1	-1	1	-1	-1	1	1	-1	1
54	1	1	-1	1	-1	1	1	-1	1	-1
55	1	1	-1	1	1	-1	-1	1	-1	-1
56	1	1	-1	1	1	1	-1	-1	1	1
57	1	1	1	-1	-1	-1	1	-1	1	1
58	1	1	1	-1	-1	1	1	1	-1	-1
59	1	1	1	-1	1	-1	-1	-1	1	-1
60	1	1	1	-1	1	1	-1	1	-1	1
61	1	1	1	1	-1	-1	-1	-1	-1	1
62	1	1	1	1	-1	1	-1	1	1	-1
63	1	1	1	1	1	-1	1	-1	-1	-1
64	1	1	1	1	1	1	1	1	1	1
65	-1	-1	-1	-1	-1	-1	-1	-1	-1	-1
66	-1	1	1	-1	0	0	-1	0	1	-1
67	-1	0	0	-1	1	1	-1	1	0	-1
68	1	0	0	0	-1	0	0	-1	1	-1
69	1	-1	-1	0	0	1	0	0	0	-1
70	1	1	1	0	1	-1	0	1	-1	-1
71	0	1	1	1	-1	1	1	-1	0	-1
72	0	0	0	1	0	-1	1	0	-1	-1
73	0	-1	-1	1	1	0	1	1	1	-1
74	0	0	-1	-1	-1	0	0	0	0	0
75	0	-1	1	-1	0	1	0	1	-1	0
76	0	1	0	-1	1	-1	0	-1	1	0
77	-1	1	0	0	-1	1	1	0	-1	0
78	-1	0	-1	0	0	-1	1	1	1	0
79	-1	-1	1	0	1	0	1	-1	0	0
80	1	-1	1	1	-1	-1	-1	0	1	0
81	1	1	0	1	0	0	-1	1	0	0
82	1	0	-1	1	1	1	-1	-1	-1	0
83	1	1	-1	-1	-1	1	1	1	1	1
84	1	0	1	-1	0	-1	1	-1	0	1
85	1	-1	0	-1	1	0	1	0	-1	1
86	0	-1	0	0	-1	-1	-1	1	0	1
87	0	1	-1	0	0	0	-1	-1	-1	1
88	0	0	1	0	1	1	-1	0	1	1
89	-1	0	1	1	-1	0	0	1	-1	1
90	-1	-1	0	1	0	1	0	-1	1	1
91	-1	1	-1	1	1	-1	0	0	0	1

Supplementary Table 4. Concentrations of 10 drugs used for QPOP in matched mCRC PDOs from CRC07, CRC08 and CRC11 at 3 dosages (-1, 0 1). IC_{15} and IC_{30} values are used for QPOP if the IC_{50} value is lower than C_{max} (Table 1). Else, 5% and 10% C_{max} values are used for QPOP.

**Drug**	**IC_0 (µM)**	**IC_{Lo} (µM)**	**IC_{Hi} (µM)**
5-FU	0	2.346	7.686
Oxaliplatin	0	0.496	0.992
Leucovorin	0	0.286	0.572
SN-38	0	0.01042	0.02195
Regorafenib	0	1.917	3.085
Pemrametostat	0	0.354	0.708
TP-064	0	7.264	9.848
Decitabine	0	0.032	0.065
Entinostat	0	0.019	0.038
Vorinostat	0	0.519	0.793

Supplementary Table 5. Parameter estimates and significance of QPOP analyses on matched CRC07, CRC08 and CRC11 PDOs. Statistical analyses were performed using sum of squares F-test (*p < 0.05; **p < 0.01; ***p < 0.001).

	ptCRC07		mCRC07		ptCRC08		mCRC08		ptCRC11		mCRC11	
	Estimate	Significance	Estimate	Significance	Estimate	Significance	Estimate	Significance	Estimate	Significance	Estimate	Significance
Intercept	1.004	*	0.904	*	0.825	*	0.883	*	0.963	*	1.255	*
5-fluorouracil	-0.122	*	0.000	**	0.001	**	0.013	**	-0.055	*	-0.019	**
Oxaliplatin	-0.256		-0.040	*	-0.243		0.004	*	-	-	-0.808	
Leucovorin	-0.383		-0.027		0.063		-0.002		-0.614		-0.087	*
SN-38	-55.747		-26.588		-7.833		-0.540		-4.743		-16.575	
Regorafenib	-0.117	*	-0.178	*	-0.138	*	-0.062	*	-	-	-0.087	*
Pemrametostat	-0.125	*	0.031		-0.050	*	-0.125	*	-0.047	*	-0.188	
TP-064	-0.004	**	-0.003	**	-0.002	**	-0.009	**	-0.008	**	-0.060	*
Decitabine	-1.065		1.050		-2.869		1.066		-5.071		-	-
Entinostat	-	-	3.269		-6.380		1.711		-8.111		-0.027	
Vorinostat	-0.419		-0.608		-0.264		0.204		-0.056	*	0.242	
5-fluorouracil:Oxaliplatin	-	-	-	-	-0.017	**	-0.018	**	-	-	-	-
5-fluorouracil:Leucovorin	-	-	-0.021	**	-	-	-	-	-	-	-	-
5-fluorouracil:SN-38	0.848		1.077		-	-	-	-	0.517		-	-
5-fluorouracil:Regorafenib	-	-	-	-	-	-	-	-	-	-	0.004	**
5-fluorouracil:TP-064	-	-	-	-	-	-	-0.001	***	-	-	0.001	***
5-fluorouracil:Vorinostat	-	-	-	-	-	-	-0.019	**	-0.015	**	-0.019	**
Oxaliplatin:Leucovorin	-0.157		-	-	-	-	-	-	-	-	-	-
Oxaliplatin:SN-38	6.919		-	-	-	-	-	-	-	-	6.042	
Oxaliplatin:Regorafenib	-	-	0.034	*	-	-	0.018	**	-	-	0.065	*
Oxaliplatin:Pemrametostat	-	-	-	-	-	-	-0.077	*	-	-	-	-
Oxaliplatin:TP-064	-	-	-	-	-0.008	**	-	-	-	-	-	-
Oxaliplatin:Decitabine	-	-	1.124		-	-	0.783		-	-	-	-
Oxaliplatin:Vorinostat	-0.090	*	-	-	-	-	0.072	*	-	-	-	-
Leucovorin:Regorafenib	-	-	0.084	*	-	-	-	-	-	-	-	-
Leucovorin:Pemrametostat	-	-	0.175		-	-	-	-	-	-	-	-
Leucovorin:TP-064	-	-	-0.014	**	-	-	-	-	-	-	-	-
Leucovorin:Decitabine	-	-	-4.694		-3.416		-3.001		-	-	-	-
Leucovorin:Entinostat	-	-	-	-	-	-	-2.582		-	-	-	-
Leucovorin:Vorinostat	-	-	0.220		-	-	0.282		-	-	-	-
SN-38:Regorafenib	4.909		2.195		2.829		1.161		-	-	2.355	
SN-38:Pemrametostat	7.194		-	-	-	-	-	-	-	-	6.287	
SN-38:TP-064	-	-	-	-	-	-	0.293		-	-	-	-

SN-38:Entinostat	-	-	-	-	-	-	68.276		-	-	-	-
SN-38:Vorinostat	-	-	5.730		-	-	-	-	-	-	-	-
Regorafenib:Pemrametostat	-	-	-	-	0.038	*	-	-	-	-	-	-
Regorafenib:TP-064	-	-	-	-	-	-	-0.003	***	-	-	-0.003	**
Regorafenib:Decitabine	0.398		-	-	-	-	-0.584		-	-	-	-
Regorafenib:Entinostat	-	-	-	-	-	-	-0.592		-	-	-0.729	
Regorafenib:Vorinostat	-	-	0.057	*	0.095	*	0.075	*	-	-	-	-
Pemrametostat:Decitabine	-	-	2.330		-	-	2.102		3.283		-	-
Pemrametostat:Entinostat	-	-	-9.711		-	-	-	-	-	-	6.055	
Pemrametostat:Vorinostat	-	-	-	-	-	-	0.313		-	-	-	-
TP-064:Entinostat	-	-	-	-	-0.196		-	-	-	-	-	-
Entinostat:Vorinostat	-	-	-	-	-	-	-1.998		-	-	-4.368	
Decitabine:Vorinostat	-	-	-2.738		-	-	-	-	-	-	-	-
5-fluorouracil^2	0.013	**	-	-	-	-	-	-	0.005	**	-	-
Oxaliplatin^2	0.237		-	-	0.324		-	-	-	-	0.479	
Leucovorin^2	0.734		-	-	-	-	-	-	0.834		-	-
SN-38^2	1175.376		555.878		-	-	-	-	-	-	-	-
TP-064^2	-	-	-	-	-	-	-	-	-	-	0.005	**
Decitabine^2	-	-	-	-	64.003		-	-	58.971		-	-
Entinostat^2	-	-	-	-	182.443		-	-	202.225		-	-
Vorinostat^2	0.581		0.561		-	-	-0.719		-	-	-	-
R_2 (QPOP)	0.948		0.922		0.892		0.946		0.826		0.897	

Supplementary Table 6. List of antibodies.

Protein target	Brand	Catalogue number	Antibody dilution	Application
CK7	Abcam	ab68459	1:200	Immunohistochemistry / Immunofluorescence
CK20	Abcam	ab76126	1:200	Immunohistochemistry / Immunofluorescence
Ki67	Sigma	AB9260	1:100	Immunohistochemistry / Immunofluorescence
CD44	Abcam	ab157107	1:200	Immunofluorescence
LGR5	Origene	TA503316	1:100	Immunohistochemistry / Immunofluorescence
Sox9	Abcam	ab92494	1:200	Immunofluorescence
EpCAM	Abcam	ab7504	1:200	Immunofluorescence
β-catenin	BD Transduction Laboratories	# 610153	1:200	Immunohistochemistry / Immunofluorescence
