# Supplementary material for: A functional personalised oncology approach against metastatic colorectal cancer in matched patient derived organoids
Source: NPJ Precis Oncol. 2024 Feb 27;8:52. doi: 10.1038/s41698-024-00543-8 (PMC10899621; doi:10.1038/s41698-024-00543-8)
Supplement: Supplementary file 2 — REPORTING SUMMARY [file 41698_2024_543_MOESM2_ESM.pdf]

nature portfolio

Corresponding author(s): Edward Kai-Hua Chow

Last updated by author(s): Jan 25, 2024

Reporting Summary

Nature Portfolio wishes to improve the reproducibility of the work that we publish. This form provides structure for consistency and transparency in reporting. For further information on Nature Portfolio policies, see our Editorial Policies and the Editorial Policy Checklist.

Statistics

For all statistical analyses, confirm that the following items are present in the figure legend, table legend, main text, or Methods section.

n/a	Confirmed	
☐	☒	The exact sample size (*n*) for each experimental group/condition, given as a discrete number and unit of measurement
☐	☒	A statement on whether measurements were taken from distinct samples or whether the same sample was measured repeatedly
☐	☒	The statistical test(s) used AND whether they are one- or two-sided<br>*Only common tests should be described solely by name; describe more complex techniques in the Methods section.*
☒	☐	A description of all covariates tested
☐	☒	A description of any assumptions or corrections, such as tests of normality and adjustment for multiple comparisons
☒	☐	A full description of the statistical parameters including central tendency (e.g. means) or other basic estimates (e.g. regression coefficient) AND variation (e.g. standard deviation) or associated estimates of uncertainty (e.g. confidence intervals)
☒	☐	For null hypothesis testing, the test statistic (e.g. *F*, *t*, *r*) with confidence intervals, effect sizes, degrees of freedom and *P* value noted<br>*Give P values as exact values whenever suitable.*
☒	☐	For Bayesian analysis, information on the choice of priors and Markov chain Monte Carlo settings
☒	☐	For hierarchical and complex designs, identification of the appropriate level for tests and full reporting of outcomes
☒	☐	Estimates of effect sizes (e.g. Cohen's *d*, Pearson's *r*), indicating how they were calculated

Our web collection on statistics for biologists contains articles on many of the points above.

Software and code

Policy information about availability of computer code

Data collection	Data was collected and exported on Microsoft Excel.
Data analysis	All statistical analyses were performed on the Prism 9 software (GraphPad), while all analyses for QPOP were performed on the proprietary software, Optim.AI ™ (Kyan Technologies).

For manuscripts utilizing custom algorithms or software that are central to the research but not yet described in published literature, software must be made available to editors and reviewers. We strongly encourage code deposition in a community repository (e.g. GitHub). See the Nature Portfolio guidelines for submitting code & software for further information.

Data

Policy information about availability of data

All manuscripts must include a data availability statement. This statement should provide the following information, where applicable:

- Accession codes, unique identifiers, or web links for publicly available datasets
- A description of any restrictions on data availability
- For clinical datasets or third party data, please ensure that the statement adheres to our policy

The phenotypic drug sensitivity data supporting the findings of this study are available upon reasonable request from the corresponding author. However, the paired patient genomic data cannot be publicly shared due to the ethical considerations from the NUH-domain-specific review board and are available from the

corresponding author upon reasonable request through collaborative investigations. The paired PDO genomic data have been deposited on the Sequence Read Archive (SRA) repository (BioProject accession number PRJNA1068196).

Research involving human participants, their data, or biological material

Policy information about studies with human participants or human data. See also policy information about sex, gender (identity/presentation), and sexual orientation and race, ethnicity and racism.

Reporting on sex and gender	No sex-based analysis was performed.
Reporting on race, ethnicity, or other socially relevant groupings	No such analysis was performed.
Population characteristics	Populations characteristic were reported in Supplementary Tables, but analysis of population characteristics was not relevant and hence not performed.
Recruitment	Primary patient material was received from National University Hospital (NUH) for the establishment of PDOs with informed consent.
Ethics oversight	Study approval from the NUH-domain-specific review board (DSRB) (NHG-DSRB Ref: 2019/00817) and National University of Singapore (NUS)-Institutional Review Board (IRB) (NUS-IRB Ref: LH-18-032E) was received.

Note that full information on the approval of the study protocol must also be provided in the manuscript.

Field-specific reporting

Please select the one below that is the best fit for your research. If you are not sure, read the appropriate sections before making your selection.

☒ Life sciences ☐ Behavioural & social sciences ☐ Ecological, evolutionary & environmental sciences

For a reference copy of the document with all sections, see nature.com/documents/nr-reporting-summary-flat.pdf

Life sciences study design

All studies must disclose on these points even when the disclosure is negative.

Sample size	No sample size calculations were performed, but we acknowledged in the manuscript that this study is a proof-of-concept study with a small sample size.
Data exclusions	No data was excluded.
Replication	All experiments were performed in triplicates, unless otherwise stated.
Randomization	NA
Blinding	NA

Reporting for specific materials, systems and methods

We require information from authors about some types of materials, experimental systems and methods used in many studies. Here, indicate whether each material, system or method listed is relevant to your study. If you are not sure if a list item applies to your research, read the appropriate section before selecting a response.

Materials & experimental systems

n/a	Involved in the study	
☐	☒	Antibodies
☒	☐	Eukaryotic cell lines
☒	☐	Palaeontology and archaeology
☒	☐	Animals and other organisms
☒	☐	Clinical data
☒	☐	Dual use research of concern
☒	☐	Plants

Methods

n/a	Involved in the study	
☒	☐	ChIP-seq
☒	☐	Flow cytometry
☒	☐	MRI-based neuroimaging

Antibodies

Antibodies used	Antibody details are included in Supplementary Table 6.
Validation	Validation for the antibodies are provided on the datasheet from the manufacturers as detailed in Supplementary Table 6.

Plants

Seed stocks	NA
Novel plant genotypes	NA
Authentication	NA
